# A role for α-Synuclein in axon growth and its implications in corticostriatal glutamatergic plasticity in Parkinson’s disease

**DOI:** 10.1186/s13024-020-00370-y

**Published:** 2020-03-30

**Authors:** Meir Schechter, Jessica Grigoletto, Suaad Abd-Elhadi, Hava Glickstein, Alexander Friedman, Geidy E. Serrano, Thomas G. Beach, Ronit Sharon

**Affiliations:** 1 Department of Biochemistry and Molecular Biology, IMRIC, The Hebrew University-Hadassah Medical School, Ein Kerem, 9112001 Jerusalem, Israel; 2 Electron Microscopy Unit, The Hebrew University-Hadassah Medical School, Ein Kerem, 9112001 Jerusalem, Israel; 3 McGovern Institute for Brain Research and Department of Brain and Cognitive Sciences, Massachusetts Institute of Technology, Cambridge, MA 02139 USA; 4 Banner Sun Health Research Institute, Sun City, AZ USA

**Keywords:** Parkinson’s disease, Corticostriatal axons, α-Synuclein, Phosphatidylinositol 4,5-bisphosphate (PI4,5P_2_), White matter tracts (WMTs), Axonal injury, Axonal growth

## Abstract

**Background:**

α-Synuclein (α-Syn) is a protein implicated in the pathogenesis of Parkinson’s disease (PD). α-Syn has been shown to associate with membranes and bind acidic phospholipids. However, the physiological importance of these associations to the integrity of axons is not fully clear.

**Methods:**

Biochemical, immunohistochemical and ultrastructural analyses in cultured neurons, transgenic mouse brains, PD and control human brains.

**Results:**

We analyzed the ultrastructure of cross-sectioned axons localized to white matter tracts (WMTs), within the dorsal striatum of old and symptomatic α-Syn transgenic mouse brains. The analysis indicated a higher density of axons of thinner diameter. Our findings in cultured cortical neurons indicate a role for α-Syn in elongation of the main axon and its collaterals, resulting in enhanced axonal arborization. We show that α-Syn effect to enhance axonal outgrowth is mediated through its activity to regulate membrane levels of the acidic phosphatidylinositol 4,5-bisphosphate (PI4,5P_2_). Moreover, our findings link α-Syn- enhanced axonal growth with evidence for axonal injury. In relevance to disease mechanisms, we detect in human brains evidence for a higher degree of corticostriatal glutamatergic plasticity within WMTs at early stages of PD. However, at later PD stages, the respective WMTs in the caudate are degenerated with accumulation of Lewy pathology.

**Conclusions:**

Our results show that through regulating PI4,5P_2_ levels, α-Syn acts to elongate the main axon and collaterals, resulting in a higher density of axons in the striatal WMTs. Based on these results we suggest a role for α-Syn in compensating mechanisms, involving corticostriatal glutamatergic plasticity, taking place early in PD.

## Background

α-Synuclein (α-Syn) is a protein known for its critical roles in the cytopathology and genetics of PD [1]. In the brain, α-Syn pathology in the form of Lewy bodies and neurites, is strongly associated with severity of the disease and propagates in an ordered and predictable regional pattern [2, 3]. Neuroanatomy studies suggest that the length and caliber of axons, together with the degree of myelination, determine neuronal vulnerability to Lewy pathology [4–6]. Specifically, susceptible neuronal cells may be projection neurons that express α-Syn and generate long and thin axon, which is poorly or unmyelinated.

Phylogenetic studies indicated a remarkable degree of α-Syn sequence conservation throughout the evolution of vertebrate branches [7, 8] and identified two homologous proteins, β-Syn and γ-Syn. Analysis of the amino acid sequence of α-Syn protein indicated the occurrence of a 11-mer repeats which make up a conserved apolipoprotein-like class-A_2_ helix [9]. These structural homologies provided the rationale for investigating the role of α-Syn associations with membrane lipids in its pathophysiology. Importantly, the phylogenetic studies also indicated the absence of α-Syn expression in invertebrates, including arthropods and cephalopods that possess a simple central nervous system [10]. Suggesting a possible involvement of α-Syn in the development of vertebrate’s brain complexity.

The cortex connects topologically with striatum through corticostriatal connections that play a central role in developing complex intentional-directed behaviors. Different regions of striatum have been associated with different cortical functions, including, emotions, cognition and motor control. Cortical glutamatergic efferent enter the striatum through striatal white matter tracts (WMTs), then make striatal synaptic contacts that influence the output nuclei of the basal ganglia [11]. In PD, degeneration of dopamine-containing neurons in the substantia nigra pars compacta (SNc), diminishes dopamine-containing innervations of the striatum and results in abnormal functioning of the striatum. Importantly, plasticity of the corticostriatal glutamatergic pathway is suggested to accommodate the loss of dopamine in early stages of the disease [12].

Collateral axon branching is a multifaceted mechanism controlled by numerous factors. One such factor is phosphatidylinositol 4,5-bisphosphate (PI4,5P_2_), a phosphorylated derivative of the membrane phospholipid, phosphatidylinositol (PI) [13]. PI4,5P_2_ is generally formed through phosphorylation by members of the type-1 phosphatidylinositol phosphate-5 kinase family (PIPKI). The three members of PIPKI family, PIPKI α, β and γ, were shown to play differential roles in neurite formation and elongation. Key proteins involved in mechanisms of axonal elongation and branching were shown to recruit members of PIPKI to this process. Interestingly, PIPKIα and PIPKIβ were shown to suppress the elongation of axons [14–17]. However, PIPKIγ was implicated in axon elongation and organization of the growth cone [18, 19].

We discover a role for α-Syn-associations with PI4,5P_2_ in elongation of axons and collaterals. In accord, we identify a remarkable increase in the density of low-diameter axons within WMTs localized to the dorsal striatum of old and symptomatic α-Syn tg mouse brains. Using axonal and synaptic markers, we demonstrate a higher density of glutamatergic axons within corticostriatal WMTs and glutamatergic terminals, in the caudate of postmortem human brains affected with PD at early stages of the disease. We suggest that α-Syn effect to increase axon outgrowth underlies the reported glutamatergic plasticity, taking place early in PD.

## Methods

### Human brains

Formalin fixed, paraffin embedded brain sections, containing the caudate and internal capsule, of advanced PD (Braak stage 5–6) and age-matched control brains, were supplied by the Multiple Sclerosis Society Tissue Bank, funded by the Multiple Sclerosis Society of Great Britain and Northern Ireland, registered charity 207,495. Additional brain sections of early PD cases (Unified stage IIa-IIb [3]) and relevant control brains were provided by the Banner Sun Health Research Institute, Sun City, Arizona, USA (Supplementary Table S1). The approval for the use of human tissue material was obtained from the Peer Review Panel of the Parkinson’s UK Brain Bank and the Brain and Body Donation Program at Sun Health Research Institute; the latter’s operations are approved by the Western Institutional Review Board (Seattle, WA, USA).

### Mice

The human PrP-A53T α-Syn tg mouse line [20] was purchased from Jackson Laboratory (Bar Harbor, ME, USA) as hemizygous; cross-bred with α-Syn^−/−^ C57BL/6JOlaHsd mice (Harlan Laboratories, Jerusalem, Israel [21]) to silence endogenous mouse α-Syn; and then bred to achieve homozygosity of the human A53T α-Syn transgene. α-Syn^−/−^ C57BL/6JOlaHsd genotype was used as control mice [21]. The PrP-A53T α-Syn tg model was shown in previous studies to develop motor disabilities and to accumulate α-Syn pathology in an age-dependent manner. That is, mice appear generally healthy and show no evidence of α-Syn pathology up to the age of 8–9 months [20, 22, 23]. However, at 12 months of age and older, the large majority of mice in the colony show signs of motor disabilities accompanied with pathogenic accumulations of α-Syn in the central nervous system. The number of sick mice grow with age and the oldest mice in the colony are ~ 16 months old. All animal welfare and experimental protocols were approved by the Committee for the Ethics of Animal Experiments of the Hebrew University of Jerusalem NIH approval # OPRR-A01–5011 (Permit number: MD-16-14,826-3).

Thy-1 hWT α-Syn mice [24, 25] were obtained from Prof. Eliezer Masliah (UCSD, USA). Control mice were non-transgenic littermates. The Thy-1 hWT α-Syn mice show early signs of learning and motor disabilities at 2–4 months of age, which worsen at 8–10 months of age [25, 26]. α-Syn pathology for the Thy-1 hWT α-Syn mice was demonstrated at 8–12 months of age [23, 25].

5XFAD and control mice [27] were bred and aged at Prof. Dan Frenkel’s laboratory (Tel -Aviv University). This mouse model shows accumulation of amyloid pathology, starting at the age of 4 months, in addition to cognitive impairment, starting at 6 months of age.

Mice were housed at a 12-h dark/light cycle and were allowed free access to food and water. This study was carried out in strict accordance with the recommendations in the Guide for the Care and Use of Laboratory Animals of the National Institutes of Health. Adequate measures were taken to minimize pain and suffering.

### Transmission electron microscopy

Mice were anesthetized and perfused in Karnovsky’s *fixative solution* (2% formaldehyde and 2.5% glutaraldehyde in 0.1 M Sodium Cacodylate buffer, pH 7.4). Mouse brains were removed and 100 μm coronal sections were obtained using a vibratome (Leica Biosystems, IL, USA). Brain sections were fixed in Karnovsky’s *fixative solution* for 2 h at room temperature and then transferred to 4 °C for an additional 24 h. Sections were washed four times with 0.1 M sodium cacodylate buffer (pH 7.3) and incubated for 1 h in 1% osmium tetroxide, 1.5% potassium ferricyanide in sodium cacodylate. Sections were then washed 4 times in the same buffer; dehydrated with graded series of ethanol solutions (30, 50, 70, 80, 90, 95%) for 10 min each; then in 100% ethanol 3 times for 20 min each; followed by two changes of propylene oxide. Brain sections were infiltrated with series of epoxy resin, (25, 50, 75, 100%) for 24 h each and polymerized in the oven at 60 °C for 48 h. The blocks were sectioned by an ultramicrotome (Ultracut E, Riechert-Jung, Ontario, Canada) and sections of 80 nm were stained with uranyl acetate and lead citrate. Sections were observed using a Jeol JEM 1400 Plus Transmission Electron Microscope and pictures were taken using a Gatan Orius CCD camera.

### Cell cultures

HEK 293 T, HeLa and SH-SY5Y cell lines were maintained in Dulbecco’s modified Eagle’s medium (DMEM) supplemented with 10% FBS; 2% L-glutamine; 1% penicillin/streptomycin, sodium-pyruvate and non-essential amino acids (Biological Industries, Beit-Haemek, Israel). SK-mel2 cells express detectable levels of endogenous α-Syn, however, these are lowered with passages. Thus, experiments were performed between weeks 2–4 from thawing a frozen aliquot. Cells were maintained in minimal essential medium (MEM; Sigma-Aldrich, Rehovot, Israel) supplemented with 10% FBS; 1% L-glutamine, penicillin/streptomycin and sodium-pyruvate. Cultures were maintained at 37 °C in a 95% air/ 5% CO_2_ humidified incubator.

### Plasmids

Costume-ready Mission shRNA were from Sigma-Aldrich. Including, shSNCA (TRCN0000272292), shCntrl and shNIR2 (TRCN0000029763), that was successfully used previously [28]; pGFP-C1-PLCδ1-PH (Addgene # 21179 [29] from Tobias Meyer); pEGFPC1-Sj-1-170 and GFP-PIPKIγ (Addgene # 22294 [30] and #22299 [31] from Pietro De Camilli); pFSy(1.1) GW (Addgene # 27232 [32] from Pavel Osten). CFP-FKBP- PIPK and Lyn-FRB.

[33]. mCherry-NIR2, a kind gift from Jen Liou (UT Southwestern Medical Center). pFSy-α-Syn was constructed by ligation of a full-length α-Syn cDNA, amplified by PCR, with pFSy(1.1) GW, following digestion with AgeI-HF / Xba-I restriction enzymes. The following primers were used for α-Syn amplification: forward: 5′-GAATCACCGGTGCCGCCACCATGGATGTATTCATGAAAG G-3′ and reverse: 5′-TAACTCTAGATTAGGCTTCAGGTTCGTAGT-3′.

### Viral production and transduction

Lentiviral particles were produced by co-transfecting HEK 293 T cells with a set of three plasmids: pCMVΔR8.91; pMD2.G; and a transfer plasmid, either pFSy(1.1) or pLKO-1-puro. Transfection was performed in 10 cm dishes (2 × 10^6^ cells) using 50 μg polyethylenimine (PEI) incubated with 12.5 μg DNA at 1:1:1 M ratio for the three plasmids. 3–4 days after transfection, the conditioning medium was collected and spun for 5 min at 1500 xg to remove cell debris, filtered through a 0.45 μm membrane and spun at 93,000 xg for 2 h, at 4 °C in a swinging-bucket rotor. Pellets containing virus particles were collected in serum-free medium and stored at − 80 °C, in aliquots. Each aliquot was thawed once, immediately before use. Virus titer was determined for each preparation following transduction of naïve SH-SY5Y cells, by quantitative PCR using specific primers either for WPRE gene (pFSy plasmids): forward 5′-CCGTTGTCAGGCAACGTG-3′ and reverse 5′-AGCTGACAGGTGGTGGCAAT-3′; or Puromycin resistance gene (pLKO-1-puro plasmids): forward, 5′-TCACCGAGCTGCAAGAACTCT-3′ and reverse primer, 5′-CCCACACCTTGCCGATGT-3′. Primer sequence for human SNCA: forward: 5′-GCAGGGAGCATTGCAGCAGC-3 and reverse 5′-GGCTTCAGGTTCGTAGTCTTG-3′; G6PD: forward: 5′- CACCATCTGGTGGCTGTTC − 3 and reverse 5′- TCACTCTGTTTGCGGATGTC − 3;

Viral transduction of cultured cells was performed by incubating the cells (1.5 × 10^6^) in FBS-free DMEM, containing viral particles and polybreane (4 μg/ml) for 6 h. The conditioning medium was then replaced with 10% FBS-supplemented DMEM. Viral transduction of primary cortical neurons was performed at 1 day in vitro (DIV) in full Neurobasal-A medium without polybreane.

### Primary cultures

Cortical cultures were prepared from cortices, dissected from a day old (P1) C57BL/6 J or C57BL/6JOlaHsd mouse brains, as described previously [34]. Cells (~ 50,000) were plated onto coverslips, pre-coated with 12.5 μg/ml poly-D-lysine (Sigma-Aldrich) in a 12-well dish. Cortical neurons were maintained in Neurobasal-A medium (Gibco, Thermo Fisher Scientific, Petah Tikva, Israel) and supplemented with 2% B-27 (Gibco, Thermo Fisher Scientific); 1% L-glutamine; 0.5% penicillin/streptomycin. To eliminate glia cells, 1 μM cytosine β-D-arabinofuranoside (Ara-C; Sigma-Aldrich) was added to the culture at 1–2 DIV. Culture medium was partially (25–50%) replaced every 4 days. Cultures were maintained at 37 °C in a 5% CO_2_ humidified incubator.

Hippocampal cultures were prepared from CA1-CA3 regions dissected from a day old (P1) WT C57BL/6 J or α-Syn^−/−^ C57BL/6JOlaHsd mouse brains as described previously [34].

Mesencephalic neurons were prepared from brains of mice at E13.5 embryos as described [35].

Electroporation of primary neurons was performed on day of preparation. Neurons were electroporated using the Amaxa Nucleofector (Lonza, Tuas, Singapore) according to manufacturer’s protocol [36]. 1.0 × 10^6^ cells were suspended in 100 μl of Ingenio electroporation solution (Mirus Bio LLC, Madison, WI, USA) containing 2.5 μg of DNA, in a nucleofection cuvette using program O-05. Cells were centrifuged to remove the electroporation medium and suspended in conditioning medium.

### Tissue punches

Mouse brains were removed, washed with cold PBS and placed on ice. A coronal segment of the brain, containing Bregma 0 - (− 3) was removed. Tissue punches (pooled from both hemispheres) were taken using a needle (1 mm) from the dorsal striatum. Punches were weighed and stored at − 80 °C until use. Tissue was homogenized by ten up-and-down strokes of Teflon Dounce homogenizer, in 10 volumes (weight/volume) of homogenization buffer containing HEPES, 20 mM; EDTA, 1 mM; MgCl_2_, 1 mM; sucrose, 0.32 M; a protease inhibitor cocktail (Sigma, Rehovot, Israel); and 1% NP-40, at 4 °C. The homogenates were centrifuged at 1500 xg for 10 min to remove cell debris. Protein samples (30 μg) were loaded on a 10% SDS-PAGE, and following electrophoresis, were transferred to a nitrocellulose membrane (Biorad, Petach Tikva, Israel). The membrane was blocked with 10% non-fat dry milk in 10 mM Tris-HCl, 150 mM NaCl, pH 8.0, containing 0.1% Tween-20 (TBST) for 1 h. The membrane was then incubated at 4 °C for 16–18 h with the indicated antibody, in TBST. For antibody details see supplementary Table S2. Immunoreactive bands were detected with HRP-conjugated secondary antibody (1:10,000). The signal was visualized with EZ-ECL (Biological Industries, Beit Haemek, Israel), scanned by a Umax Magic Scan (Eastman Kodak, Rochester, NY, USA) and analyzed for density of each signal using UN-SCAN-IT GEL 3.1 software (Silk Scientific, Orem, UT, USA).

### FACS

Analysis was performed as previously described [37] with some modifications. Cells were suspended and washed in clear DMEM; followed by 20 min fixation in 2% (v/w) paraformaldehyde at 4 °C; and permeabilization in 0.2% saponin in 1% BSA (w/v) for 15 min at 4 °C. Cells were then incubated with anti α-Syn antibody (MJFR1, 1:2000) and anti PI4,5P_2_ antibody (1:200, see supplementary Table S2) for 90 min at 4 °C with gentle rolling; washed and probed with the respective secondary antibody for 30 min at room temperature. FACS analysis in SK-mel2 cells was performed 7–14 days following viral transduction. During this time effective α-Syn knockdown was confirmed using rt-PCR and Western blotting. HEK 293 T cells were analyzed by FACS 48 h from transfection. Analyses were performed using BD LSRFortessa Cell Analyzer, equipped with 5 lasers (355, 405, 488, 561 and 640 nm) and FLOWJO, LLC software. Mock-GFP, Sj-1 or PIPKIγ expression were directly detected at 488 nm based on a GFP-tag. Each experiment also included adequate compensation controls. In each experiment a control, consisting of cells grown and processed in parallel, treated with ionomycin [38] (10 μM) for 7 min at room temperature, was included. Gating was based on FSC, SSC and positive immunoreactivity for the relevant proteins (i.e., α-Syn, Sj-1 and PIPKγ). A total of 3000–4000 gated cells were counted in each experiment unless indicated differently.

### PI4,5P_2_ detection by PH-PLCδ1-GFP biosensor

HeLa cells were grown on cover slides coated with poly-D-Lysine, in 12-well plates. Cells were co-transfected with PH-PLCδ1-GFP and either WT, A53T or K10,12E α-Syn expressing plasmids, using JetPRIME transfection reagent polyplus (Tamar, Rehovot, Israel). Forty eight hours post transfection, cells were incubated with 50 μg/ml Alexa-647 Concanavalin (Con) A (molecular probes, Invitrogen, Rehovot, Israel) in DMEM, at 37 **°**C for 10 min; washed in cold serum-free DMEM; and fixed with 4% paraformaldehyde for 10 min, on ice. Cells were then washed one more time and permeabilized with 0.2% Triton X-100 in blocking solution (1.5% BSA in PBS) for 5 min at room temperature. Cells were incubated with anti α-Syn antibody, C20 (Santa Cruz, Dallas TX, US) at 1:500 dilution, overnight at 4 °C, followed by a secondary ab. Membrane to cytosolic signal ratio of PH-PLCδ1-GFP was calculated using the NIS-Element AR Analysis 4.20.02 64-bit software (Nikon, Agentek, Tel Aviv, Israel). Membranes were defined by the ring-shaped ConA signal around the cell and differentiated from the cytoplasm of the cells.

### Immunocytochemistry (ICC)

Primary neurons grown on cover slips, at 4 or 14 DIV were gently washed with warm HBSS (Biological Industries, Beit-Haemek, Israel), fixed with 2% PFA for 20 min at room temperature and permeabilized with 0.5% saponin in 1% (w/v) BSA for 30 min, at room temperature. Cover slips were then incubated for 2 h at room temperature with the indicated primary antibodies (Supplementary Table S2) in 1% (w/v) BSA, followed by 3 washes in PBS, 5 min each. Slides were then incubated with appropriate secondary ab, washed and mounted in vectashield® mounting medium (Vector-labs, Burlingame, CA USA).

### Immunohistochemistry for mouse brain sections

Paraffin-embedded, coronal mouse brain sections (6 μM) were processed for immunostaining as previously described [39]. The antibodies used are listed in supplementary Table S2. Images were acquired using a Zeiss LSM 710 Axio Observer confocal Z1 laser scanning microscope, equipped with an argon laser 488, Diode 405–430 laser and HeNe 633 laser. Images at Fig. 3g; Fig. 5a and supplemtary 2B were captured at higher resulation using Nikon’s A1R+ confocal microscope, equipped with an ultrahigh-speed resonant scanner and high-resolution digital galvano scanner, with four laser unit LU-N4S. Per each experiment, the exciting laser, intensity, background levels, photo multiplier tube (PMT) gain, contrast and electronic zoom were maintained constant. Antibody specific background was subtracted. The focus of each picture was obtained by choosing the plane with greatest fluorescent signal.

### Immunohistochemistry for human brains

Slides containing formalin fixed, paraffin embedded brain sections of advanced PD and controls, immunoreacted with anti SMI-32 ab [40] or anti α-Syn antibody (BD Transduction Labs), were provided by the Multiple Sclerosis Society Tissue Bank. Otherwise, slides were processed for immunostaining as previously described [39]. The antibodies used are listed in supplementary Table S2. Images were acquired using a Nikon Ti Eclipse motorized inverted microscope with DIC, phase epi-fluorescence optics and Perfect Focus System (PFS). Equipped with a Nikon DS-Fi1 color CCD camera and NIS-Elements image acquisition software. Fluorescence images were acquired using a Zeiss LSM 710 Axio Observer confocal Z1 laser scanning microscope (as above). All images were taken using the same settings, and on the same day. The specific signal inside WMTs was quantified per area and normalized to the nonspecific signal outside of WMTs. Quantification of SMI-32, vGlut1 and TH immunoreactivity in the caudate were performed based on 6–10 fields per brain at × 20 magnification (Image J). Fields were chosen randomly. Images were taken and analyzed blindly to tissue classifying information.

### Quantifications

Quantifications were performed blinded to treatments. To reduce experimental error, comparisons were made within slides that were processed and analysed in parallel. Image series were analyzed with Image Pro Plus 6.3 (Media Cybernetics, Bethesda, MD, USA) or Fiji (Image J). An average value was calculated for each animal, followed by calculation of the average for the group. Data presented in percent of control cells when including different immunostaining events. Quantitation of the signal localized specifically to neurites was performed with Image J as recently described [41]. Determination of neurite and axon length, number and length of collateral branches (longer than 15 μm) was done by tracing an axon including its collateral branches, starting from the cell body throughout, using the segmentation plugin for neurite tracer in Image J.

### Statistics

Comparisons between two groups were performed by two-tailed ttest. Additional comparisons were performed by one-way ANOVA and Dunnett test for correction for multiple comparisons (Prism 7). Data presented as mean ± SD or mean ± SE, as indicated. Significant differences were considered with *P* ≤ 0.05.

## Results

### Higher density of thinner axons in striatal WMTs of α-Syn tg mouse brains

**Fig. 1:**
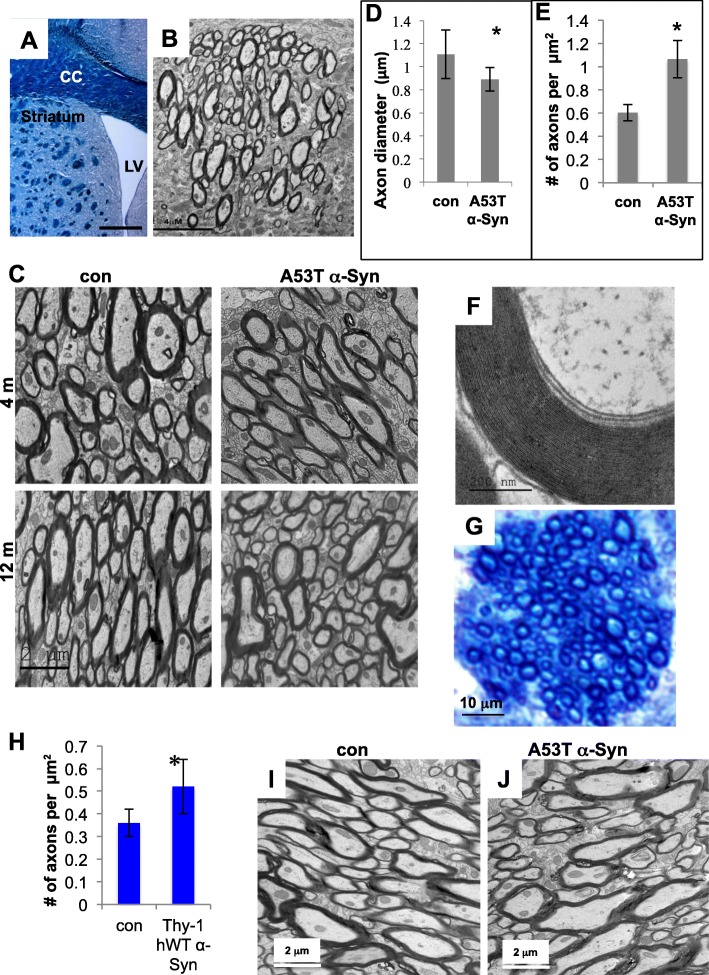
Ultrastructure of cross-sectioned axons in white matter tracts (WMTs). **a** Lower magnification of a coronal mouse brain section, stained with Luxol Fast blue (Sigma-Aldrich), showing the striatum. CC, corpus callosum; LV, lateral ventricle; WMTs shown as dark spots over the lightly stained striatal grey matter. Bar = 1 mm. **b** Cross section of a WMT in the dorsal striatum, consisting of myelinated axons. Image obtained by transmission electron microscope (TEM). Bar = 4 μm. **c** TEM images of coronal brain sections containing WMTs from A53T α-Syn and control mouse brains, at 4 months (healthy mice) or 12 months of age (symptomatic). Bar = 2 μm. **d** Bar graph showing the diameter (in μm) of cross-sectioned axons in WMTs. **e**. The number of axons per μm^2^ area of WMTs (axon density). Mean ± SE *n* = 4 brains, 5–8 WMTs per brain. **f** Intact myelin ultrastructure of a cross-sectioned axon in a WMT of 12 months old A53T α-Syn tg mouse brain. Bar = 200 nm. **g** A semi-thick A53T α-Syn mouse brain section (1 μm), stained with methylene blue (Sigma-Aldrich), showing cross-sectioned axons in a WMT. Bar = 20 μm. **h** Graph showing axon density in WMTs of Thy-1 hWT α-Syn and control mouse brains at 10–12 months of age. Determined in semi-thick sections (1 μm) stained with methylene blue. Mean ± SE of *n* = 4 brains, 8–10 WMTs per brain. **i** and **j** TEM images showing sagittal brain sections across the axons in corpus callosum of 12–14 months old A53T and age-matched control (C57BL/6JOlaHsd) mouse brains

To investigate a potential role for α-Syn in axon integrity we analyzed the ultrastructure of cross-sectioned axons, localized within striatal WMTs, in coronal sections of A53T α-Syn tg and control mouse brains [20]. We reasoned that the anatomical organization of the axons within the bundles and the relevance of the brain area to the disease could best fit our focus of investigation. The tissue block was set to contain the dorsal striatum, just underneath the corpus callosum, using the size of the lateral ventricle as a reference for tissue position (Fig. 1a,b). Axon diameter and the density of axons were determined within WMTs of similar size (~ 1.5–4 × 10^− 10^ m^2^) and similar location at 2–4 months of age, representing healthy, fully myelinated mouse brains [42] and at the age of 12–14 months, representing symptomatic mice [20, 22] (Fig. 1c-e). A significantly lower diameter was detected for axons of 12–14 months old A53T α-Syn tg (0.89 ± 0.1 μm) than in age-matched control mouse brains (1.1 ± 0.2 μm). Mean ± SE of *n* = 4 brains, 8–10 WMTs per brain; *P* < 0.01, ttest. Surprisingly, the number of myelinated axons per μm^2^ within WMTs was significantly higher in the A53T α-Syn tg (1.06 ± 0.16) than in control brains (0.60 ± 0.07). Mean ± SE of *n* = 4 brains, 8–10 WMTs per brain; *P* < 0.05, ttest. In accord with our recent report [23], no overt myelin ultrastructure or pathology was detected (Fig. 1f). That is, large axons, with a standard number and structure of lamellae were commonly detected in WMTs of the A53T α-Syn brains. However, compared with the control brains, a higher number of thinner axons, which are only sparsely myelinated were detected in the A53T α-Syn brain sections (Fig. 1c). Of note, differences in axon diameter or density between control and A53T α-Syn tg brains at young, 2–4 months of age were not statistically significant.

Similar results, indicating a higher density of axons within striatal WMTs, were detected in a second α-Syn tg mouse line, the Thy-1 hWT α-Syn mice [24, 25]. Semi-thick brain sections (1 μm) from 10 to 12 months old Thy-1 hWT α-Syn and control, age-matched non-transgenic littermates, were stained with methylene blue for visualization of myelin sheath (Fig. 1g,h). Comparing between WMTs of similar size and similar location (as above), we determined 0.52 ± 0.12 and 0.38 ± 0.06 myelinated axons per μm^2^ in Thy-1 hWT α-Syn and control mouse brains, respectively. Mean ± SE of *n* = 4 brains, 6–8 WMTs per brain. *P* < 0.05, ttest.

The EM blocks containing coronal brain sections positioned at dorsal striatum, including the corpus callosum, were cut one more time in a position corresponding to sagittal brain sections (Fig. 1i,j). The sections, across the axons in corpus callosum of 12–14 months old A53T α-Syn and control mouse brains, revealed a high variability in axon diameter and axon density. No differences in the ultrastructure of axons were detected in the corpus callosum*.*

### Longer axons and collaterals in primary cultures of mouse neurons expressing α-Syn

**Fig. 2:**
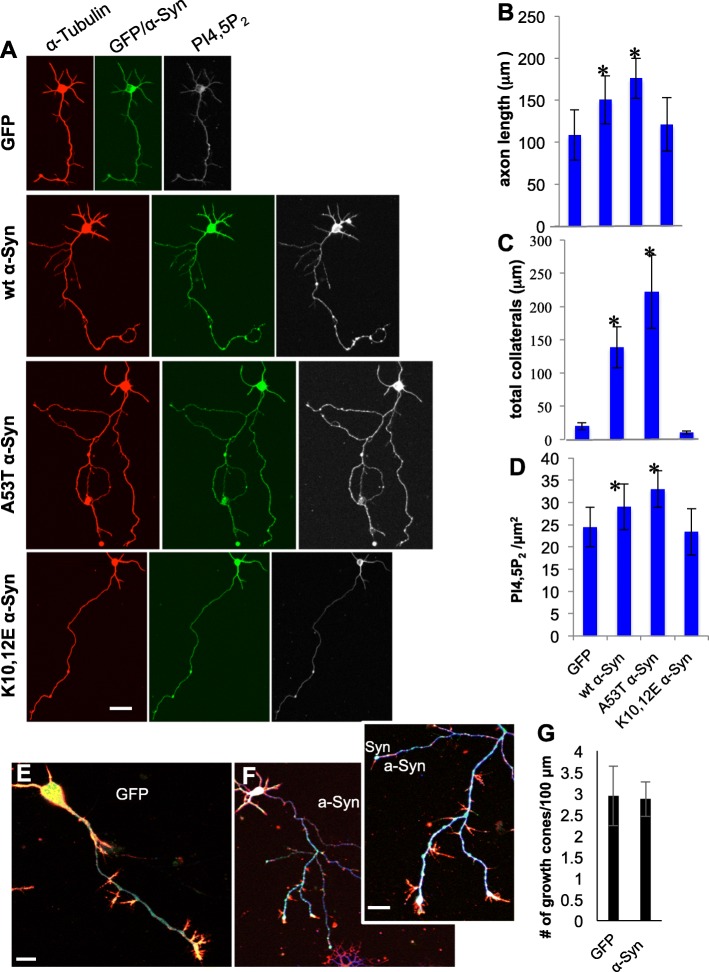
Elongated axons and collaterals in primary cortical neurons expressing α-Syn associate with higher levels of phosphatidylinositol 4,5-bisphosphate (PI4,5P_2_). **a** Primary cortical cultures from α-Syn^−/−^ (C57BL/6JOlaHsd) mouse brains, virally transduced either with WT α-Syn, A53T α-Syn, K10,12E α-Syn, or a mock-GFP vector. Cells were fixed at 4 DIV and immunoreacted with antibodies against α-Syn (MJFR1, green), α-tubulin (red) and PI4,5P_2_ (white). Direct fluorescence was captured for GFP (green). Bar = 25 μm. **b** Graph showing the average axon length (in μm); **c** Total length of collaterals per axon (in μm); and **d** PI4,5P_2_ levels within the axon and its collaterals (per μm^2^ area) quantified by Fiji (Image J) program. Mean ± SE; *n* > 22 cells; *, *P* < 0.05. **e** A primary cortical neuron as in (**a**), transduced with a mock-GFP (green); immunoreacted with anti GAP-43 (blue) and stained with rhodamine-phalloidin (red). Bar = 10 μm **F.** A neuron as in (**e**) expressing WT α-Syn and immunoreacted with anti α-Syn (MJFR1, green) and anti α-tubulin (blue) abs, and stained with rhodamine-phalloidin (red). Shown an entire cell and a zoom on axon collaterals. Bar = 5 μm. No differences in number of growth cones per axon (in μm) were detected. **g** Graph bar showing quantitation of growth cones per μm axon in WT α-Syn and GFP expressing neurons. Mean ± SD of *n* = 12–15 cells

To investigate a potential involvement of α-Syn in elongation and/or branching of axons we studied primary cultures of cortical neurons from α-Syn^−/−^ mouse brains. The cultures were transduced to express either human WT or A53T α-Syn, or a synthetic K10,12E α-Syn mutation generated by replacing two positively charged Lysine residues, within the KTKEGV repeat domain, with negatively charged Glutamic acid residues. In a previous study, this mutation was shown to interfere with α-Syn binding to membrane phospholipids [43]. Control cells expressed a mock-GFP vector. Cells were fixed at 4 DIV and immunoreacted with antibodies against α-Syn, α-tubulin and the acidic phosphoinositide, PI4,5P_2_. The average length of the primary axon in control cortical neurons, transduced with the mock virus (in μm) was 108.50 ± 29.9. Significantly longer axons were measured for WT α-Syn expressing (150.43 ± 28.6) and furthermore for A53T α-Syn expressing neurons (175.97 ± 23.8). Axon length in neurons expressing the K10,12E mutation (121.2 ± 31.8) was not different than in control cells. Mean ± SE; *n* > 22 cells; *P* < 0.05, one way ANOVA (Fig. 2a,b).

A dramatic effect on the total length of collaterals, extending from the main axon, was observed. The total length of collaterals (per axon) was ~ 6.5 folds higher in WT α-Syn and ~ 10 folds higher in A53T α-Syn than control neurons. In contrast, the length of collaterals in neurons expressing K10,12E α-Syn did not differ from control cells (Fig. 2c, mean ± SE; n > 22 cells; *, *P* < 0.05; one way ANOVA).

Importantly, similar to A53T α-Syn, expression of A30P α-Syn in primary mouse cortical neurons resulted in longer axons and longer collaterals compared with WT α-Syn expressing neurons (Supplementary Fig. S1E). Moreover, α-Syn effects to enhance axon outgrowth were similarly detected in primary mouse hippocampal as well as mesencephalic neurons (Supplementary Fig. S1).

### Altered PI4,5P_2_ levels in α-Syn expressing neurons

Phosphoinositides are a group of acidic phospholipids and PI4,5P_2_ is implicated in axonal growth [13, 18, 19]. Attempting to find out whether α-Syn associations with membrane phospholipids and its preference for acidic phospholipids [44] may play a role in its effects to enhance axon outgrowth, we co-immunoreacted the primary cortical neurons with anti PI4,5P_2_ ab (Fig. 2a). PI4,5P_2_ levels were determined per axon area (μm^2^, Fig. 1d) by ICC. Setting PI4,5P_2_ levels in control axons at 100%, we detected significant ~ 118% and ~ 135% higher PI4,5P_2_ levels in WT and A53T α-Syn expressing axons, respectively. The A30P mutation in α-Syn similarly increased PI4,5P_2_ levels over WT α-Syn (Supplementary Fig. S1E). However, expression of K10,12E α-Syn mutation had no detectable effects on PI4,5P_2_ levels (*n* = 22–24 cells, *P* < 0.05, one-way ANOVA). α-Syn expression in hippocampal neurons resulted in similar increases in axonal PI4,5P_2_ levels. That is, PI4,5P_2_ levels in hippocampal axons (per μm^2^) expressing WT or A53T α-Syn were 122 and 131% higher (respectively) than in axons expressing a mock virus, set at 100% (Supplementary Fig. S1D).

### α-Syn expression does not alter the number of growth cones per axon

The increases in axon density demonstrated in Fig. 1 could potentialy result from increases in the number of growth cones per μm axon. To assess growth cones, we co-immunoreacted cultured cortical neurons at 4 DIV, expressing either mock-GFP or WT α-Syn, with an anti GAP-43 antibody, a marker for growth cones and phalloidin, a marker for filamentous actin (Fig. 2e). A parallel immunoreaction included anti α-tubulin and phalloidin (Fig. 2f). Closely similar numbers of growth cones per μm axon were found for α-Syn expressing (2.85 ± 1.0) and mock-GFP expressing cells (2.93 ± 1.7) using the different antibodies (Mean ± SD, *n* = 12–15 cells). Suggesting no effect for α-Syn expression on the number of growth cones per axon (Fig. 2g).

### α-Syn regulates PI4,5P_2_ levels

**Fig. 3:**
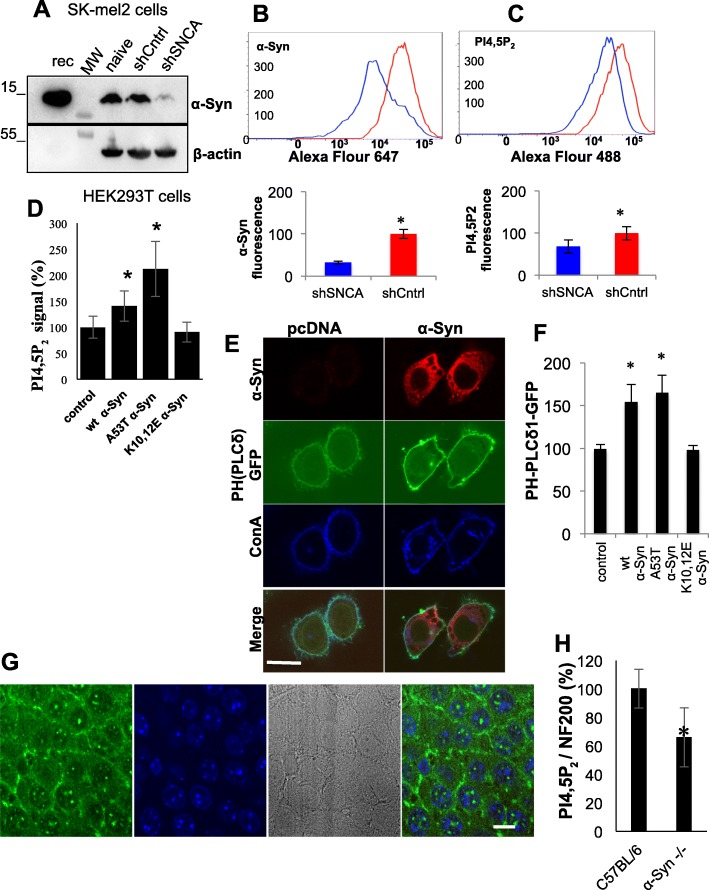
α-Syn expression regulates PI4,5P_2_ levels. **a** SK-mel2 cells transduced with viral particles expressing either sh-SNCA or a control (sh-Cntrl). 3–7 days post transduction, samples of lysed cells (80 μg protein) were analyzed by Western blotting for α-Syn expression using MJFR1 antibody. **b** SK-mel2 cells transduced as in (**a**) and analyzed by FACS for α-Syn, using anti α-Syn antibody MJFR1; and **c** using anti PI4,5P_2_ antibody. Mean ± SE, *n* = 4000 cells; *, *P* < 0.05, ttest. **d** HEK 293 T cells were transfected either with WT α-Syn, A53T or K10,12E α-Syn mutations, or mock transfected. 48 h post transfection, PI4,5P_2_ levels were determined by FACS, using anti PI4,5P_2_ antibody. Cells were gated based on positive signal for α-Syn detected by MJFR1 antibody. Mean ± SE, *n* > 3000 cells; *, *P* < 0.05, one way ANOVA. **e** HeLa cells, co-transfected with WT α-Syn and PH-PLCδ1-GFP. Control cells transfected with the mock plasmid together with PH-PLCδ1-GFP. Cells were incubated with Alexa-647 Concanavalin (Con) A for visualization of plasma membrane (PM) and processed for ICC with anti α-Syn antibody, C20. Showing the co-localization of PH-PLCδ1-GFP and 647-ConA signals (direct fluorescence). Bar = 10 μm. **f** Calculated plasma membrane to cytosol ratio of the PH-PLCδ1-GFP signal in HeLa cells, co-expressing the indicated constructs. Mean ± SE of *n* = 15-25 cells. *, *p* < 0.05, one way ANOVA. **g** High magnification of paraffin embedded mouse brain section containing the hippocampus, analyzed by IHC and immunoreacted with anti PI4,5P_2_ ab (green). Section stained with DAPI (blue). Nomarski image (gray) shown to demonstrate PI4,5P2 signal on PM. Bar = 10 μM. **h** Primary cortical neurons from WT C57BL/6 or α-Syn −/− (C57BL/6JOlaHsd) at 14 DIV. Cultured neurons analyzed by ICC and immunoreacted with anti NF-200 and anti PI4,5P_2_ abs. Bar graph shows signal ratio of PI4,5P_2_ to NF200. Mean ± SD, *n* = 5 fields, consisting of > 10 neuronal cell bodies each; *, *P* < 0.05, t-test

In a series of experiments, we assessed the associations of α-Syn with PI4,5P_2_ and the specificity of these associations. Silencing endogenous α-Syn expression in SK-mel2 cells with shRNA resulted in ~ 70% lower α-Syn levels relative to control cells, transduced with a scrambled shRNA (set at 100%; Fig. 3a). In accord, PI4,5P_2_ levels, determined by FACS were ~ 34% lower in shSNCA compared with shCntrl expressing cells (Fig. 3b,c). Mean ± SE; *n* = 4000 cells; *P* < 0.05, ttest. PI4,5P_2_ levels were next determined by FACS in HEK 293 T cells, transfected to express α-Syn (Fig. 3d). A significantly higher PI4,5P_2_ signal was detected in WT α-Syn (140.7 ± 15.5%) and A53T α-Syn (211.9 ± 33.2%) expressing cells than in control cells (set at 100%). Mean ± SE, *P* < 0.05, one way ANOVA. However, PI4,5P_2_ levels in cells expressing the K10,12E α-Syn mutation were not different than control cells (Fig. 3d).

Similar results were obtained using a PH-PLCδ1-GFP molecular indicator of PI4,5P_2_ levels. HeLa cells were transfected to express WT, A53T, K10,12E α-Syn or a mock vector, together with a plasmid expressing PH-PLCδ1-GFP. Cells were incubated with 647-concanavalin A (ConA) to mark the plasma membrane (Fig. 3e,f). The relative fluorescence intensity of PH-PLCδ1-GFP in plasma membrane to cytosol was set at 100% for the mock plasmid expressing cells. Significant 158.9 ± 37% and 169.0 ± 38% higher values were detected for WT and A53T α-Syn expressing cells, respectively. No effect for the K10,12E α-Syn mutation on PH-PLCδ1-GFP signal was detected. Mean ± SE of 3 independent experiments; *n* = 15–25 cells in each experiment, *p* < 0.05, one-way ANOVA.

To find out if endogenous mouse α-Syn similarly enhances PI4,5P_2_ levels in vivo, we immunoreacted paraffin embedded, coronal brain sections from 2 months old α-Syn^−/−^ (C57BL/6JOlaHsd) and age-matched WT C57BL/6 mice with anti PI4,5P_2_. We found a significantly lower signal in α-Syn ^−/−^ (61.8%) than control WT mice (set at 100%, Fig. 3g). *P* < 0.01, ttest. *N* = 5 mouse brains in each genotype. Importantly, PI4,5P_2_ signal appeared in the nuclei and on the PM surrounding the cell body, supporting the specificity of the antibody-detected signal. The effect of mouse endogenous α-Syn on PI4,5P_2_ levels was next tested in primary hippocampal neurons at 14 DIV prepared in parallel from WT C57BL/6 and α-Syn^−/−^ mouse brains (Fig. 3h). Cultured neurons were co-immunoreacted with anti PI4,5P_2_ and anti neurofilament (NF-200) antibodies. Normalizing the signal obtained for PI4,5P_2_ to the signal detected for NF-200, we detected a significant lower PI4,5P_2_ signal in hippocampal neurons from α-Syn^−/−^ (~ 66%) than C57BL/6 mouse brains (set at 100%). Mean ± SD, *n* = 5 fields, consisting of > 10 cells; *P* < 0.05, ttest.

To confirm the specificity of the PI4,5P_2_ signal we detect using anti PI4,5P_2_ ab in cortical neurons, we determined PI4,5P_2_ signal depletion following phospholipase C (PLC) activation. Primary cortical neurons from α-Syn^−/−^ (C57BL/6JOlaHsd) mouse brains were treated with carbachol (1 mM at 5 DIV), a muscarinic agonist that activates PLC [45]. Control cells were conditioned and treated in parallel but without the drug. Cells were fixed and immunoreacted with anti PI4,5P_2_ ab (Supplementary Fig. S2A). PI4,5P_2_ signal in the carbachol treated neurons (25%) was dramatically lower than the signal detected in control neurons, treated with DMSO solvent (set at 100%; *P* < 0.01, ttest). Importantly, in images captured at higher magnification, the loss of PI4,5P_2_ signal is clearly detected on the PM of the cell body and throughout the axon, supporting specificity of the detected PI4,5P_2_ signal (Supplementary Fig. S2B). Similar results, showing loss of PI4,5P_2_ signal following PLC activation in primary cortical neurons were obtained following activation of the muscarinic receptors with acetylcholine (10 μM) and pilocarpine (9.6 μM).

As an additional approach to study PI4,5P_2_ signal specificity, we expressed the rapamycin-induced translocatable CF-PIPK construct, which consists of an active PIPKIγ, a flourosence CFP and a FKBP domain [33]. In HEK293T cells, CF-PIPK fluorescence was largely in the cytosol and moved to the PM with the addition of rapamycin. In accord with the localization of CF-PIPK, the detectable PI4,5P_2_ signal was intracellular or on the PM, respectively (Supplementary Fig. S2C).

### α-Syn effect to elongate the main axon and collaterals requires PI4,5P_2_

**Fig. 4:**
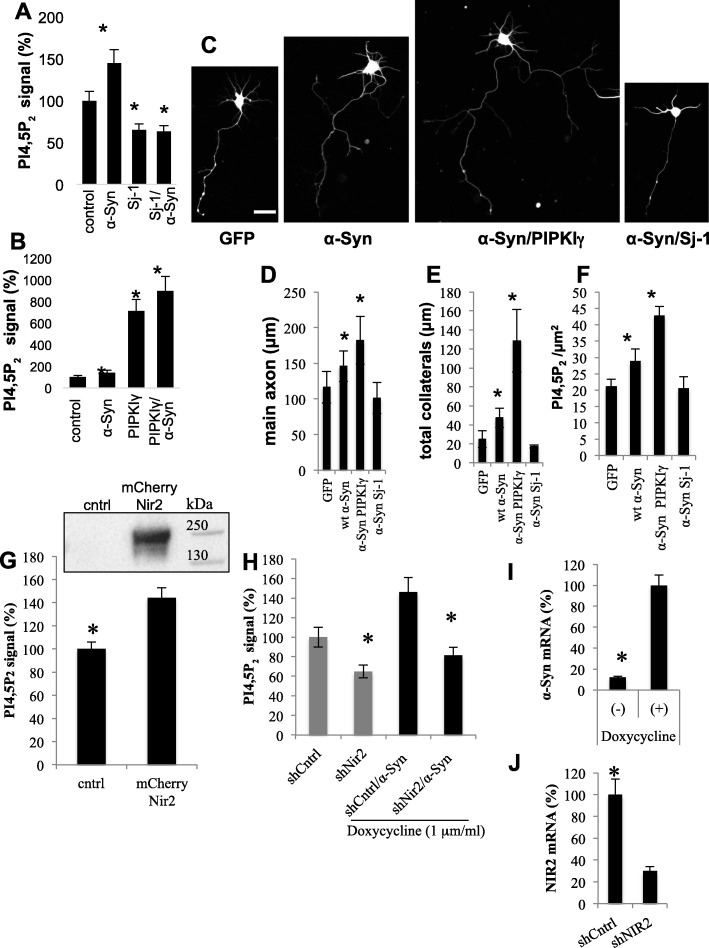
α-Syn effect to elongate axons and collaterals is mediated through PI4,5P_2_. **a** PI4,5P_2_ levels determined by FACS, in HEK 293 T cells, following 48 h from cell transfection with α-Syn and/or Sj-1 phosphatase, as indicated. Mean ± SD of two independent experiments, n > 3000 cells in each treatment; *P* < 0.05, one-way ANOVA. **b** PI4,5P_2_ levels determined by FACS in HEK 293 T cells transfected with α-Syn and/or PIPKIγ as indicated. Mean ± SD of two independent experiments, n > 3000 cells in each treatment; *P* < 0.05, one-way ANOVA. **c** Primary cortical cultures from α-Syn^−/−^ mouse brains, electroporated at day of preparation to co-express WT α-Syn together either with PIPKIγ-GFP, Sj-1-GFP or mock-GFP expressing vectors (as indicated). Cultures were fixed and immunoreacted with anti α-Syn MJFR1 ab and α-tubulin. GFP signal was captured by direct fluorescence. α-tubulin signal is shown. Bar = 25 μm. **d** The average length of axons (in μm). **e** Total length of collaterals per axon (in μm); and **f** PI4,5P_2_ levels within the axon and its collaterals (per axon area) quantified by Fiji program. Mean ± SE; *n* > 25 cells; *, *P* < 0.05 one-way ANOVA. **g** HEK 293 T cells transfected to express mCherry-Nir2 or mock transfected (cntrl). Western blot showing mCherry-Nir2 signal detected with anti Nir2 ab (Abcam). PI4,5P_2_ levels determined by FACS. Mean ± SE, n > 3000 cells; *, *P* < 0.05, ttest. **h** Tet-on inducible SH-SY5Y cells for inducible α-Syn expression were infected with viral vectors expressing either shNIR2 or shCntrl (Mission, Sigma-Aldrich). α-Syn expression induced with doxycycline (1 μM/ml) or non-induced. PI4,5P_2_ levels determined by FACS. Mean ± SD of *n* = 3 different experiments, > 3000 cells in each treatment. **i** Quantitative PCR (qPCR) detection of α-Syn following its induced expression in SH-SY5Y cells without and with the addition of doxycycline for 72 h. α-Syn mRNA levels normalized to the levels of G6PD gene detected in the same sample. **j** Quantitative detection of NIR2 expression by qPCR in these cells

To investigate the potential involvement of PI4,5P_2_ in α-Syn-dependent axonal outgrowth, we tested the effects of synaptojanin-1 (Sj-1), a PI4,5P_2_*5*-*Phosphatase* or PIPKIγ to alter PI4,5P_2_ levels in HEK 293 T cells expressing α-Syn (Fig. 4a,b). In cells co-expressing α-Syn and Sj-1 the increase in PI4,5P_2_ levels, associated with its expression (e.g., ~ 132% increase), was denied and PI4,5P_2_ levels were below the levels of control cells, expressing the mock plasmid. In accord, a dramatic ~ 700% increase in PI4,5P_2_ levels was detected with PIPKIγ expression and an additive effect of ~ 20% was observed in cells co-expressing α-Syn and PIPKIγ (Fig. 4b). Mean ± SD of *n* > 3000 cells; *P* < 0.05, one-way ANOVA.

We next co-expressed α-Syn either with Sj-1or PIPKIγ in primary cortical neurons from α-Syn^−/−^ mouse brains by electroporation. Control neurons expressed WT α-Syn together with a mock plasmid or a GFP expressing vector. Cultures were fixed and immunoreacted with antibodies against α-Syn, α-tubulin and PI4,5P_2_. The expression of Sj-1, PIPKIγ and GFP were visualized directly based on their GFP tag. Similar to the results above (Fig. 2), longer axons and longer collaterals were measured for α-Syn expressing cells (Fig. 4c-e). Specifically, the length of the main axon (in μm) was 116.5 ± 22.1 in control cells and 145.7 ± 21.7 in WT α-Syn expressing cells. Co-expression of WT α-Syn together with PIPKIγ further increased axon length (182.1 ± 33.6) and co-expression of WT α-Syn together with Sj-1, eliminated the elongative effect of α-Syn (101.0 ± 21.8) with a mean value that is lower than control cells expressing the mock-GFP plasmid (Fig. 4c-d). The total length of collaterals per axon in WT α-Syn expressing cells (47.1 ± 6.7 μm) was longer than control cells (24.8 ± 7.9 μm). Further increase in length of collaterals was observed with PIPKIγ (128.6 ± 19.3 μm) yet, α-Syn effect to increase the length of collaterals was eliminated when co-expressed with Sj-1 (17.1 ± 8.1 μm; Fig. 4c, e, mean ± SD of *n* > 20 cells, *, *P* < 0.05; one way ANOVA).

Quantifying PI4,5P_2_ levels in the axons, we found a significant increase with WT α-Syn expression (~ 128%) and furthermore in cells expressing α-Syn and PIPKIγ (~ 205%). However, PI4,5P_2_ levels in axons expressing α-Syn and Sj-1 (~ 98%) were not different than control cells (100%; Fig. 4f). Similar results, demonstrating the importance of PI4,5P_2_ levels for α-Syn-mediated elongation of axons and collaterals were obtained in primary mesencephalic neurons (Supplementary Fig. S1E-G). Based on these findings we conclude that the mechanism through which α-Syn acts to elongate the axons is dependent on PI4,5P_2_.

### The regulatory role of α-Syn on PI4,5P_2_ levels is Nir2-dependent

To test the mechanism through which α-Syn increases cellular PI4,5P_2_ levels, we tested the potential involvement of Nir2-expression. HEK293T cells were transfected to express mCherry-NIR2 or a mock (control) plasmid. 72 h post DNA transfection, PI4,5P_2_ levels were determined by FACS. In line with a previous report [28], over-expressing mCherry-Nir2 in HEK 293 T cells increased PI4,5P_2_ levels (Fig. 4g). To find out whether Nir2 expression is required for α-Syn-mediated increases in PI4,5P_2_ levels, we utilized SH-SY5Y cells, that inducibly express α-Syn under a Tet-On control [46]. Cells were infected to silence NIR2 expression using a viral vector expressing shNIR2. Control cells were infected with shCntrl. Five days post infection, doxycycline (1 μM) was added to the cells to activate α-Syn expression. PI4,5P_2_ levels were determined by FACS following 3 days of incubation with doxycycline. The efficacy of shNIR2 to silence NIR2 expression was tested by qPCR and the levels were found to be 70% lower than in cells infected with the scrambled shRNA (set at 100%, Fig. 4j). α-Syn levels of expression were ~ 25 folds higher with doxycycline (Fig. 4i). The results show that doxycyclin enhanced α-Syn expression resulted ~ 146% higher PI4,5P_2_ signal. However, the effect of doxycxline induced α-Syn-expression on PI4,5P_2_ signal was abolished when cells were infected to silence NIR2 expression (Fig. 4h, mean ± SD of *n* = 3 different experiments; *n*= > 3000 cells in each treatment).

### Higher PI4,5P_2_ levels in striatal WMTs of α-Syn tg mouse lines

**Fig. 5:**
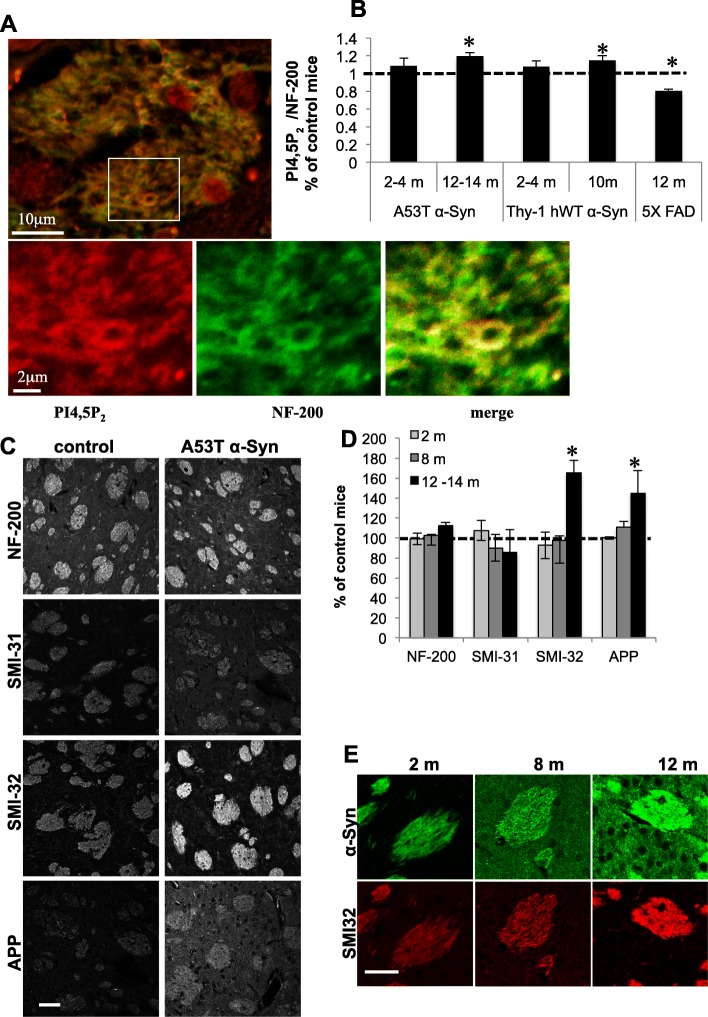
Differences in WMTs between α-Syn transgenic and control mouse brains. **a** Paraffin embedded coronal brain section (6 μm) from A53T α-Syn tg mouse, immunoreacted with anti NF-200 (green) and anti PI4,5P_2_ (red) antibodies. Showing a WMT and the localization of the immunoreactivity to axon membrane. **b** Graph showing the immunoreactive signal ratio obtained for PI4,5P_2_ and NF-200 within WMTs (per area) for A53T α-Syn or Thy-1 hWT α-Syn tg mouse models, presented as percent of age- and genotype- matched control mice set at 100% (represinted by a vertical line). Mean ± SE, *n* = 4 brains, *, *p* < 0.05, one-way ANOVA. **c** Paraffin embedded coronal brain sections (6 μm) of A53T α-Syn and control (α-Syn^−/−^, C57BL/6JOlaHsd) mouse brains at 12 months of age, containing the dorsal striatum, immunoreacted either with antibodies against axonal markers SMI-32, SMI-31 and NF200 or anti APP antibody, a marker for axonal damage. Bar =50 μm. **d** Quantification of the immunoreactivity obtained as in (**c**) in WMTs of A53T α-Syn tg and control (α-Syn^−/−^, C57BL/6JOlaHsd) mouse brains, at 2, 8 and 12–14 months of age. Vertical line represents age-matched control mice, set at 100% for each of the tested antibodies. Mean ± SE; *n* = 5 mouse brain. *, *P* < 0.05, one-way ANOVA. **e** Consecutive brain sections (as in **c** and **d**) co-immunoreacted with syn303 anti α-Syn and anti SMI-32 antibodies. Bar = 20 μM

To draw a line between the findings in mouse brains, showing a higher density of axons within WMTs and the findings in primary neurons, showing an effect for α-Syn to enhance axon outgrowth, we next determined PI4,5P_2_ levels in striatal WMTs of α-Syn tg and control brains. Paraffin embedded brain sections of healthy young and symptomatic old mice of two mouse models, the A53T α-Syn and the Thy-1 hWT α-Syn mice were analyzed by immunohistochemistry (IHC). The respective age and genotype-matched control mice were analyzed in parallel (see methods). The position of the sections was set as above (Fig. 1a). Brain sections were co-immunoreacted with PI4,5P_2_ and NF-200 antibodies (Fig. 5a). PI4,5P_2_ levels were normalized to NF-200 signal, obtained within WMTs (per area). Setting PI4,5P_2_ to NF-200 ratio of age and genotype- matched control mouse brains at 100%, we detected ~ 109% and significant ~ 120% higher ratio in 2–4 and 12–14 months-old A53T α-Syn mouse brains, respectively. Similar results, showing ~ 107% and significant ~ 115% higher ratio were detected in WMTs of 2–4 and 10 months-old Thy-1 hWT α-Syn mouse brains, respectively (Fig. 5b; mean ± SE of *n* = 4 brains; *P* < 0.05, one-way ANOVA). In a control experiment, PI4,5P_2_ to NF-200 signal ratio was determined in WMTs of 12 months-old 5XFAD mice, modeling Alzheimer’s disease. Importantly, in contrast to the PD mouse models, the results show a significant lower (~ 70%) ratio in WMTs of old 5XFAD mice than in control brains (100%) [27]. Mean ± SE of *n* = 4 brains; *P* < 0.05. Together, specific higher PI4,5P_2_ levels per axon were detected in striatal WMTs of old, symptomatic, α-Syn tg mouse lines.

### Evidence for axonal injury within WMTs of α-Syn tg mouse brains

To find out whether the effects of α-Syn to increase axon density within WMTs are associated with its toxicity, we analyzed brain sections of A53T α-Syn at 2, 8 and 12–14 months, and age-matched control mice by IHC. Brain sections were immunoreacted with an anti SMI-32 antibody, which recognizes the non-phosphorylated epitopes on the neurofilament proteins and known for its immunoreactivity with corticostriatal axons [47]. In addition, the sections were tested for SMI-31 immunoreactivity, which recognizes phosphorylated neurofilament. Consecutive brain sections were immunoreacted with an anti amyloid precursor protein (APP) antibody, as a marker for axonal injury [48]. The signal obtained within WMTs for each of these markers, in each of the tested age groups, was set at 100% for control mice. No differences were detected up to 8 months of age (Fig. 5d). In line with evidence for increases in axonal density within WMTs (Fig. 1), we detected a significant 165 ± 18% higher SMI-32 immunoreactive signal in the α-Syn tg mouse brains. Interestingly, the significantly 144 ± 17% higher APP signal, detected in WMTs of 12–14 months old A53T α-Syn mouse brains, indicates the occurrence of axonal damage (Fig. 5 c,d; mean ± SD of *n* = 5 mouse brains). In accord with the occurrence of axonal damage, we detected an age-dependent increase in α-Syn immunoreactivity, determined with syn303 antibody, within SMI-32-positive striatal WMTs and also in striatal matrix (Fig. 5e).

### Plasticity of corticostriatal WMTs of human brains at early stages of PD

**Fig. 6:**
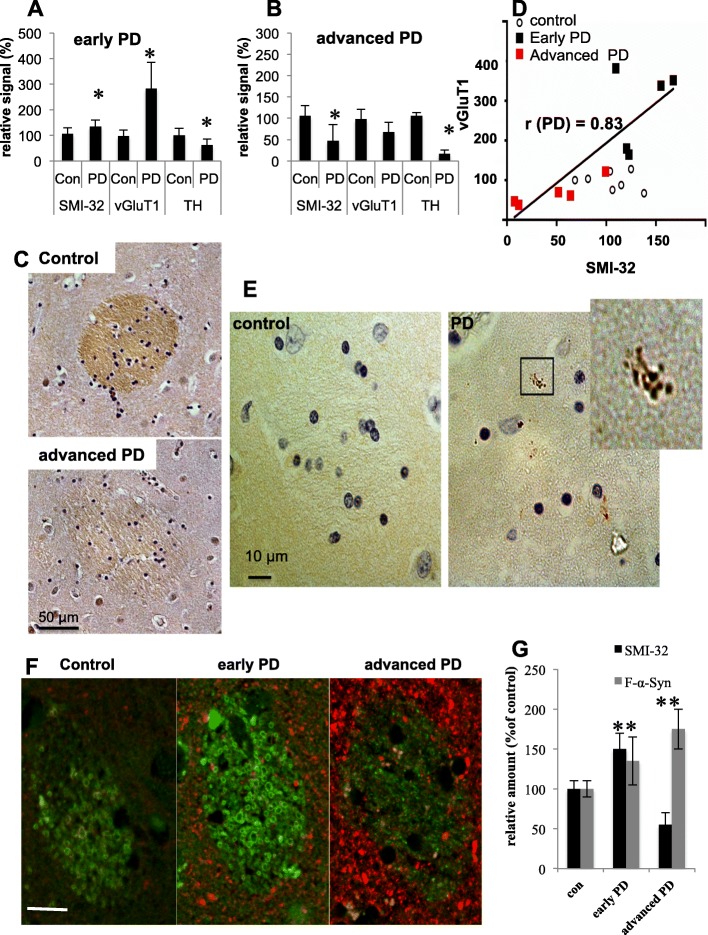
Axonal plasticity in corticostriatal connections at early stages of PD **a**. SMI-32, vGluT1 and TH immunoreactive signals localized to the caudate of early PD (unified stage IIa and IIb; and control brains. *N* = 5 each). **b** A graph (as in **a**) showing advanced PD (Braak stages 5–6; *n* = 5) and control brains (*n* = 7). Mean ± SD of 5–7 fields. *, *P* < 0.05, ttest. **c** IHC of caudal WMTs in a control (Male, 89 years) and an advanced PD brain (Male, 82 years, Braak stage 6), immunoreacted with anti SMI-32 antibody. **d** Positive correlation (Pearson’s *r* value =0.83) between the immunoreactive signals obtained for vGluT1 and SMI-32 for PD cases. **e** A caudal WMT probed for α-Syn pathology by IHC. A control brain (male, 84 years) and an advanced PD (male, 75 years, Braak stage 5) brain. **f** IHC of caudal WMTs in a control brain (male, 84 years) an early PD brain (male, 72 year, unified stage IIb) and an advanced PD brain (Male 75 years, Braak stage 5). Brain sections co-immunoreacted with anti filament-α-Syn and SMI-32 antibodies. Bar = 20 μm. **g** Filament-α-Syn and SMI-32 signals as in (**f**). Mean ± SD of *n* = 10–15 WMTs

To study the corticostriatal glutamatergic connections in PD brains, we determined SMI-32 signal in caudal WMTs and Vesicular glutamate transporter 1 (vGluT1) signal in corticostriatal terminals, at early PD (unified stages IIa and IIb [3]) and advanced PD (Braak stages 5–6 [2]). We reasoned that if glutamatergic plasticity indeed occurs in PD, then it is more likely to be detected at early rather than advanced disease stages. The results show a significant 135 ± 24.8% higher SMI-32 signal in caudal WMTs of early PD cases (*n* = 5) compared with control brains (*n* = 7). Mean ± SD; *P* = 0.03, ttest (Fig. 6a). In contrast, a dramatic 46.9 ± 38.1% lower SMI-32 signal was detected in advanced-PD cases (*n* = 5) compared with control brains (*n* = 7). Mean ± SD; *P* = 0.01, ttest (Fig. 6b,c). In accord, vGluT1 signal was considerably ~ 283 ± 87% higher in early PD (*n* = 5, *P* = 0.007, ttest) and 67.4 ± 32.8% lower in advanced PD (*n* = 5, *P* = 0.05, ttest) compared with control brains (*n* = 7; Fig. 6a,b and supplementary Fig. S3).

TH immunoreactivity was similarly determined in the caudate to test dopaminergic synapses in these human brains. TH signal was lower (61.9 ± 8.6%) in early PD and considerably lower (16.5 ± 9.7%) in the tested advanced PD cases (*P* < 0.05, ttest; Fig. 6a,b and supplementary Fig. S3).

To find out whether SMI-32 immunoreactivity within WMTs correlates with vGluT1 immunoreactivity at corticostriatal terminals, we co-immunoreacted PD and control brain sections with SMI-32 and vGluT1 antibodies. A strong positive correlation between SMI-32 and vGluT1 signals (Pearson’s *r* value =0.83) was detected in the caudate of PD brains (*n* = 10). However, no correlation could be detected for the control brains (Pearson’s *r* value − 0.21; *n* = 7). The results therefore suggest that at early PD, increases in glutamatergic axons within WMTs correlate with increases in glutamatergic terminals in the caudal matrix. However, with progression of disease, both, glutamatergic axons and terminals are degenerated (Fig. 6d).

**Fig. 7:**
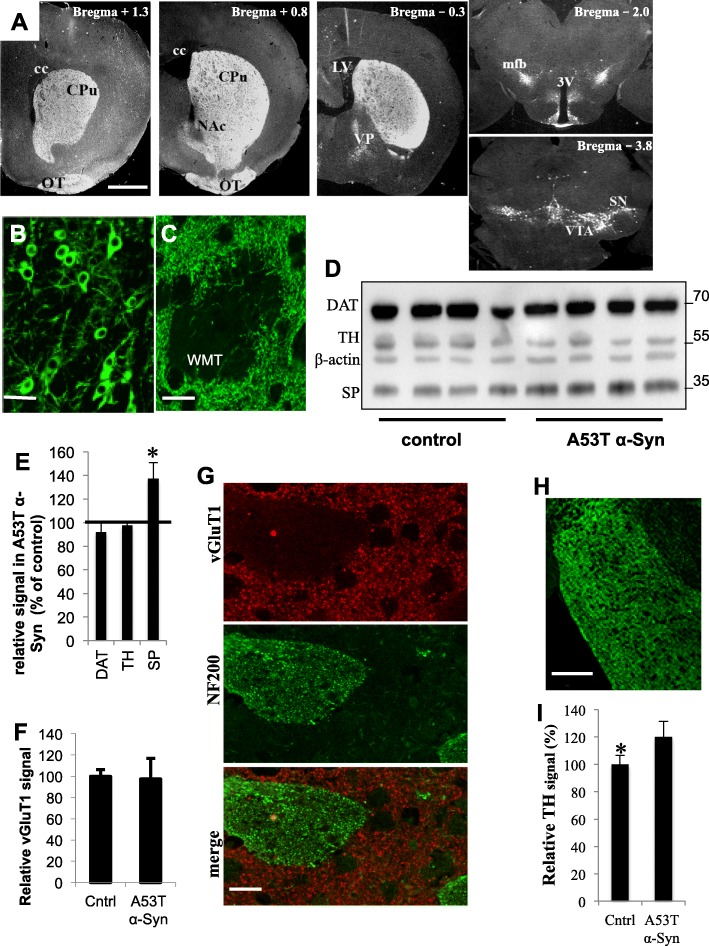
The dopaminergic system in A53T α-Syn tg mouse brains. **a** Coronal brain sections (6μm, bregma + 1.3- (− 3.8)) of A53T α-Syn tg mouse (14 months), immunoreacted with anti Tyrosine hydroxylase (TH) antibody. CC, corpus callosum; CPu, caudate putamen; LV, lateral ventricle; mfb, medial forebrain bundle; NAc, nucleus accumbens; OT, olfactory tubercle; VP, ventral pallidum; VTA, ventral tegmental area. 3 V, third ventricle. Bar = 500 μm. B-C. Higher magnification of brain sections (as in **a**). **b** SNc, Bar = 25 μm. **c** Striatal WMT. Bar = 20 μm. **d** Protein samples (30 μg) of striatal homogenates from 12 to 14 month old A53T α-Syn tg and age matched α-Syn −/− control mouse brains analyzed by Western blotting. Blot immunoreacted with antibodies for dopamine transporter (DAT); Tyrosine hydroxylase (TH); β-actin; and synaptophysin (SP). A representative blot out of two. *N* = 5 brains in each genotype. **e** A graph showing quantitation of blots in (**d**), values normalized to β-actin levels in the same sample. Vertical bar represents control brains, set at 100%. **f** Quantitative values of Western blot as in (**d**) reacted with anti vGluT1 ab. **g** Immunohistochemistry (IHC) of paraffin embedded mouse brain section co-immunoreaced with anti vGlut1 (red) and anti NF-200 (green) abs. Bar = 25 μm. **h** IHC of a paraffin embedded section containing the olfactory tubercle from an A53T α-Syn tg mouse brain at 12 month of age, co-immunoreaced with anti TH ab. **i** Quantification of immunoreactivity obtained by IHC in the olfactory tubercle. Paraffin-embedded brain sections of A53T α-Syn tg and control 12–14 months old mice, immunoreacted with anti TH antibody as in (**h**). Mean ± SD of *n* = 4 mouse brain. *, *P* < 0.05, ttest. Bar = 100 μm

Together, the analysis in human brains at early stages of PD validate the occurrence of corticostriatal plasticity within WMTs, localized to the caudate and the findings indicating higher density of SMI-32-positive glutamatergic axons in WMTs of α-Syn tg mouse brains.

Evidence for α-Syn pathology was commonly detected within caudal WMTs of advanced PD brains. α-Syn pathology, detected in cross-sectioned axons within WMTs, consists mostly of Lewy neurites and observed as α-Syn –positive granulated signal. The densely hematoxylin-stained glia cells within WMTs were devoided of Lewy pathology (Fig. 6e). Importantly, similar to the finding in the α-Syn tg mouse brains (Fig. 5e) pathogenic forms of α-Syn protein, detected with an anti filament α-Syn antibody [49] were abundantly detected within WMTs and matrix early in PD, side by side with the increase in SMI-32 immunoreactivity (Fig. 6f,g). Further increases in pathogenic α-Syn were detected at advanced PD, however, these were accompanied with degeneration and loss of SMI-32 immunoreactive axons (Fig. 6f,g). Finally, we assessed the diameter of axons within WMTs of early PD and control cases. A high degree of variability in axon diameter within a specific WMT (0.09–6.8 μm) was detected. We reasoned that the high variability in axon diameter between WMTs within the same brain section denies meaningful comparisons between the groups.

### The mouse model partly recapitulates the human disease

We thought to assess the degree in which the mouse model recapitulates alterations in dopaminergic and glutamatergic terminals, and the severity of disease. In line with the original description of this A53T α-Syn tg mouse line [20] TH-immunoreactivity detected by IHC, appeared highly similar between 12 and 14 months old A53T and age-matched control mice (Fig. 7a). No differences in TH-immunoreactivity were detected in the SNc (Fig. 7b), WMTs or striatum (Fig. 7c).

The expression levels of TH, dopamine transporter (DAT) and synaptophysin, a marker for synaptic terminals, were next determined by a quantitative Western blotting. Brain tissue punches containing the dorsal striatum of 12–14 months old mouse brains were homogenized to yield a total homogenate immediately after dissection. Protein samples of striatal homogenates (30 μg protein) were analyzed and normalized to β-actin levels detected on the same blot (Fig. 7d, e). The Western blot results confirm the finding above, indicating no differences between the tested mouse genotypes for TH and DAT levels in the striatum. However, synaptophysin levels were significantly higher in striatal homogenates of A53T α-Syn (137%) than in control brains (set at 100%). Mean of *n* = 5 brains in each genotype, *P* < 0.05, ttest. Supporting increases in axons and synaptic terminals.

vGluT1 signal was quantified in the dorsal striatum, in paraffin-embedded, coronal brain sections of 12–14 months old A53T α-Syn tg and control mouse brains, by IHC. In addition, vGluT1 signal was assessed in tissue punches containing the dorsal striatum by Western blotting. The results show no differences in vGluT1 levels between mouse genotypes (*n* = 5–7 mouse brains in each genotype).

Interestingly, TH immunoreactive signal in the olfactory tubercle, which is innervated by dopaminergic neurons residing in the ventral tegmental area (VTA) and is part of the ventral striatum, was significantly 121 ± 15% higher in the aged A53T α-Syn than in control mouse brains, set at 100% (Fig. 7h, i). Mean ± SD of *n* = 4 brains in each genotype, *P* < 0.05, ttest.

Together, the results in the mouse brains do not show evidence for actual loss of dopaminergic axons or alterations associated with glutamatergic terminals. At the age 12–14 month, the transgenic mouse colony, which expresses the human mutant A53T α-Syn and backcrossed to the C57BL/6JOlaHsd α-Syn −/− mouse genotype, recapitulates characteristic features of the human disease, including accumulation of α-Syn pathology and evidence for axonal injury, side by side with evidence for axonal growth, however, with no apparent loss of dopaminergic axons.

## Discussion

**Fig. 8:**
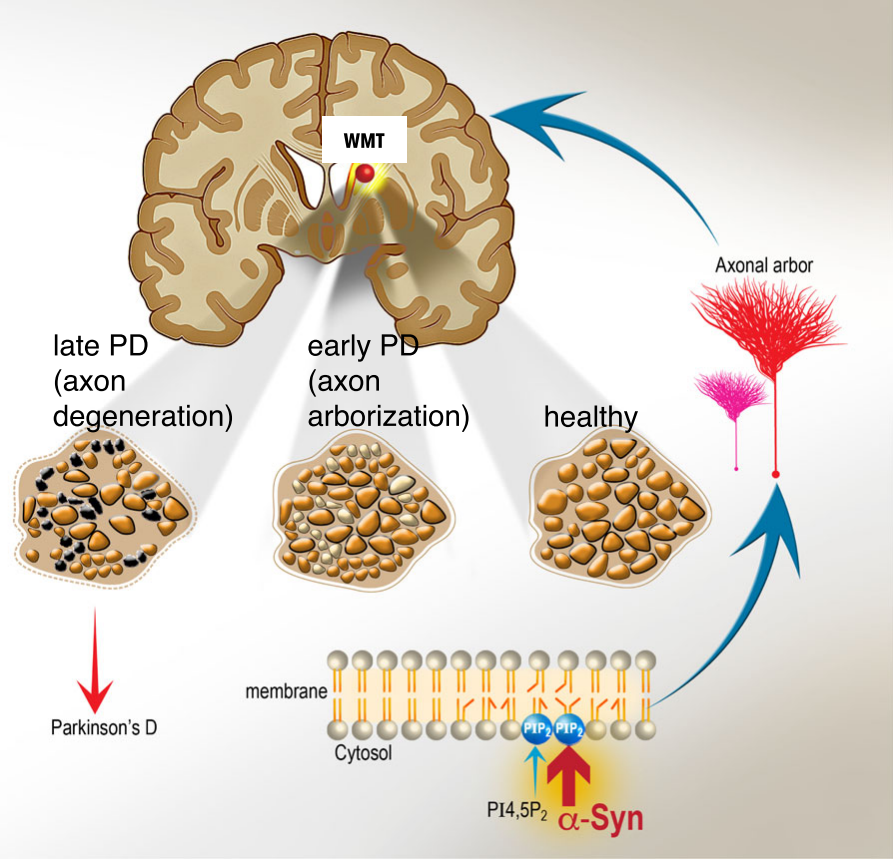
Illustrative synopsis. α-Syn enriches axon membrane with the acidic phosphoinositide PI4,5P_2_ to facilitate axon outgrowth and arborization. In brains affected with PD, over expression or mutations in α-Syn increase axon density at early stages of the disease and enhance glutamatergic plasticity in caudal white matter tracts (WMTs). However, with disease progression, accumulation of damage is leading to degeneration and loss of axons within WMTs

We demonstrate a role for α-Syn in mechanisms of axonal growth and plasticity, and relate the finding to pathogenic mechanisms in PD. Our results show that α-Syn elongates the main axons and collaterals in primary neurons, with a stronger effect for the PD- associated A53T α-Syn mutant over WT α-Syn. The involvement of α-Syn in axon outgrowth is mediated through its associations with membrane PI4,5P_2_ phosphoinositide. In brains of mice transgenic for α-Syn, we detect a higher density of axons, within striatal WMTs of old and symptomatic mice. The finding, obtained in two different α-Syn tg mouse lines, suggests an involvement of α-Syn in mechanisms of plasticity. In accord, in human PD brains we detect evidence for a higher axon density within corticostriatal, glutamatergic, WMTs at early stages of the disease. The increases in corticostriatal plasticity fit with previous indications for the occurrence of compensatory glutamatergic mechanisms in PD. These compensatory mechanisms are suggested to follow dopaminergic denervation, which underlies presentation of characteristic disease symptoms. However, side by side with enhanced plasticity at early PD, we also detected accumulation of α-Syn toxicity. With disease progression and further loss of dopamine, the corticostriatal glutamatergic WMTs degenerate (see Fig. 8 for illustrative summary).

Early studies have shown that α-Syn preferentially binds acidic phospholipids [44]. In this study, we found that α-Syn regulates PM levels of the acidic phosphoinositide, PI4,5P_2_. We suggest that the mechanism through which α-Syn regulates PI4,5P_2_ levels involves Nir2 activity. Nir2 supports the maintenance of PI4,5P_2_ pool on the PM. It acts at endoplasmic reticulum (ER)-PM contact sites [28, 50] to transfer a newly synthesized phosphatidyl inositol (PI) from the ER to the PM in exchange for phosphatidic acid (PA), in a mechanism that is tightly coupled with the hydrolysis of PI4,5P_2_ by phospholipase C (PLC). The higher levels of PI4,5P_2_ on PM of α-Syn expressing axons may facilitate outgrowth and arborization through phosphorylated GAP-43 [51–53]. GAP-43 phosphorylation at Ser41 was shown to regulate actin filament length by increasing stabilization/polymerization of F-actin in a PI4,5P_2_ -dependent mechanism [54].

The results link increases in axonal PI4,5P_2_ levels with α-Syn toxicity in PD. One obvious limitation of studying phosphoinositides in human brains is their reported instability accounted for the differences in post mortem intervals [55]. Because of this concern we have not assessed PI4,5P_2_ levels in the post mortem human brains. Based on our findings in brains of mice modeling PD, we suggest a model for α-Syn toxicity in PD that involves increases in PI4,5P_2_, which leads to elongation of axons and collaterals, and in parallel, enhances the accumulation of axonal damage. The mouse model successfully recapitulates the increases in glutamatergic, SMI-32 positive, axon density that is detected in the PD brains. However, a drawback of this study is the lack of evidence for alteration in PI4,5P_2_ in the human brains.

With regards to the experiments performed with human brains, the early and advanced PD cases were obtained from two different sources. A plausible concern is that the disease stage-dependent differences we detect may result from differences in protocols of post mortem tissue processing and/or post mortem intervals. It is important to emphasize that control cases in this study were obtained from both resources.

Parkinson’s disease is caused due to the loss of dopamine producing nerve cells [56]. The predominantly vulnerable dopaminergic neurons in PD are the dopaminergic neurons that reside in the SNc and innervate the dorsal striatum. Whereas the dopaminergic neurons in the VTA, which innervate the ventral striatum, are less affected [56–60] and were shown to resist α-Syn toxicity [61]. A characteristic feature of the dopaminergic neurons of the SNc is a complex axonal arbor with an exceptionally high number of synapses in the striatum. This feature enables broad connectivity and neuroplasticity [62], and on the other hand, may underlie their specific vulnerability in PD, attributed to a high energetic burden [63]. The findings herein, support a role for α-Syn in axon outgrowth and arborization.

The A53T α-Syn tg mouse model [20] was backcrossed to the C57BL/6JOlaHsd α-Syn −/− genotype to silence endogenous α-Syn. In its original description [20], the TH-positive neurons of this mouse model appeared unaffected and in our analysis, no overt loss in TH immunoreactivity could be detected (Fig. 7). Absence of evidence for actual loss of dopaminergic neurons was reported also for an α-Syn tg rat model [64]. We suggest that the lack of evidence for TH-positive axon loss in the dorsal striatum of A53T α-Syn tg mouse brains may represent a steady state between two opposing mechanisms taking place simultaneously in the aged transgenic mouse brain, namely, axon growth and axon degeneration.

Of relevance, compensatory mechanisms have been suggested to take place at early stages of PD. Surviving dopaminergic neurons appear to go through functional changes aimed at preserving dopamine availability [65]. Experiments in animal models performed following acute chemical-induced lesion in the SNc [12], demonstrated compensatory axonal branching in association with improvement in animals’ motor performances. Additional, non-dopaminergic mechanisms, are activated to support the changes in dopamine homeostasis [65, 66]. The loss of dopamine appears to disrupt a functional interplay between dopamine and glutamate in brains affected with PD [67–70]. It has been reported that glutamatergic transmission to the striatum is increased in PD [71]. This plasticity of the glutamatergic system in response to the loss of dopamine includes increases in synaptic strength and corticostriatal glutamatergic transmission at the remaining synapses [72, 73]. Glutamatergic terminals in striatum may represent either thalamic or corticostriatal axons. Utilizing vGluT1, a marker for corticostriatal terminals and vGlut2, a marker for thalamostriatal terminals [74–77], it was previously reported that the corticostriatal terminals are increased in the putamen of PD brains [71]. Increases in vGluT1 terminals were also detected in the striatum of chronically MPTP-treated parkinsonian monkeys [78]. In line with these reports, our findings show higher striatal vGluT1 levels at early stages of PD. We now suggest that the mechanism through which glutamatergic synapses respond to the denervation of dopaminergic axons involves plasticity of corticostriatal connections, attributed to α-Syn’ effects to enhance axon outgrowth.

Two missense mutations in the sac domain of the PI4,5P_2_–5-phosphatase Sj-1, R258Q and R459P, were recently discovered in patients affected with autosomal recessive form of early-onset Parkinsonism [79–81]. Importantly, a knock-in mouse carrying the R258Q mutation showed axonal degeneration that was selectively observed in dopaminergic axons in the dorsal striatum [82]. These recent reports support a role for phosphoinositides (PIP)s in mechanisms of PD. Of relevance, α-Syn over expression was shown to associate with impaired cytoskeleton [83, 84]; neurite outgrowth and integrity [85]; and membrane trafficking [86]. A common denominator shared by these mechanisms is the involvement of PIPs. It is plausible that differences in metabolism of PIPs, between model systems, may explain the high variability in reported effects of α-Syn in neurite outgrowth and integrity [85, 87], or in membrane trafficking [86].

Our understanding of the sequence of pathogenic events leading to PD is incomplete. It is agreed that loss of nigrostriatal dopaminergic inputs to the dorsal striatum is the central cause for the cardinal motor features of PD. However, the pathophysiology of PD extends beyond the SNc, where the dopaminergic neurons reside. An ordered and predicted disease propagation path, which affects the peripheral nervous system and propagates to the central nervous system, fits well with Braak hypothesis [2, 56, 88, 89]. This hypothesis suggests that Lewy pathology in the central nervous system (CNS) propagates in a caudal to -rostral direction, starting at the lower brainstem and the olfactory system at early stages and spreading out to the neocortex at late stages of the disease [2, 56, 89, 90]. However, side by side with the growing support, it is important to also consider evidences that do not directly line up with Braak hypothesis [3]. A recently described viewpoint suggests a different and complementary sequence of events for PD pathogenesis. According to which, the neocortex is not necessarily a final stage of a bottom-up sequence, rather, it is involved early in disease, initiating a top-down sequence that may disturb the vulnerable dopaminergic neurons. Involvement of the neocortex in disease mechanisms may explain the paradox of focal onset of motor signs in PD [91]. However, a top-down progression is not supported by autopsy series with large numbers of PD and normal aging subjects [3]. With relevance to this debate, we show degeneration of corticostriatal, SMI-32 positive WMTs, at advanced stages of the disease, along with accumulation of characteristic PD pathology. However, our results also show alterations in corticostriatal, SMI-32 immunoreactive WMTs, early in PD when loss of caudal TH is apparent. This involvement of the cortical glutamatergic connections at early stages of the disease may be critical for understanding the potential involvement of the neocortex in focal onset of motor signs in PD.

## Conclusions

α-Syn is involved in the regulation of plasma membrane levels of PI4,5P_2._ The increases in PI4,5P_2_ levels enhance axon arbor. However, excessive growth of the axons is associated with accumulation of damage and is implicated in mechanisms of Parkinson’s disease (Fig. 8).

## Supplementary information

**Additional file 1.** Table S1. Post mortem human brains. Table S2. list of antibodies.

**Additional file 2 **Supplementary Fig. S1. α-Syn expression enhances axonal growth in primary hippocampal, mesencephalic and cortical neurons. A. Primary hippocampal cultures from α-Syn^−/−^ (C57BL/6JOlaHsd) mouse brains, virally transduced either with WT α-Syn, A53T α-Syn, K10,12E α-Syn, or a mock-GFP vector. Cells were fixed at 4 DIV and immunoreacted with antibodies against α-Syn (MJFR1, green), α-tubulin (white) and PI4,5P_2_ (red). Direct fluorescence was captured for GFP (green). Bar = 25 μm. B. Graph showing the average length of the main extension designated as axons (in μm); C. Total length of collaterals per axon (in μm); and D. PI4,5P_2_ levels within the main axon and its collaterals (per μm^2^ area) quantified by Fiji (Image J) program. Mean ± SE; *n* > 17 cells; *, *P* < 0.05; Bar = 5 μm. E. Cortical neurons cultured and treated as in Fig. 2, expressing A30P α-Syn mutation, WT α-Syn or GFP (mock). Mean ± SE; *n* > 15 cells; *, *P* < 0.05. F-G. Primary mesencephalic neurons at 4 DIV from α-Syn^−/−^ mouse brains. Neurons were virally transduced to co-express α-Syn and a mock plasmid, α-Syn and PIPKIγ or α-Syn and Sj-1. Control neurons expressed a mock GFP plasmid, Cultured neurons immunoreacted (as in A) with anti TH antibody to verify dopaminergic properties, indicated ~ 90% positivity. F. Average length of the main extension designated as axons (in μm); G. Total length of collaterals per axon (in μm); and H. PI4,5P_2_ levels within the main axon and its collaterals (per μm^2^ area) quantified by Fiji (Image J) program. Mean ± SE; *n* > 20 cells; *, *P* < 0.05. Supplementary Fig. S2. Specificity of PI4,5P_2_ signal. A. Specificity of PI4,5P_2_ signal is detected by ICC in primary neurons, following activation of muscarinic receptors with carbachol (Sigma Aldrich) and PLC-mediated hydrolysis of PI4,5P_2_. Primary cortical neurons at 5 DIV, treated with 1 mM carbachol for12 minutes. Bar = 20 μM. B. Higher magnification of a cortical neuron (as in A), demonstrating PI4,5P_2_ signal on PM surrounding cell body. HEK 293 T cells transiently expressing the rapamycin-induced translocatable CFP-PIPK kinase. CFP-PIPK fluorescence is either cytosolic or on the PM in DMSO or rapamycin (respectively) treated cells. CFP-PIPK (blue) is directly detected at 485 nm and PI4,5P_2_ detected by ICC with anti PI4,5P_2_ ab (red). Bar = 10 μM. Supplementary Fig. S3. vGluT1 and TH immunoreactivity in control and PD brains. Slides containing caudate of a control (Male 85 years, 3 h PMI) and an early PD case (male 83 years, 2 h PMI, Unified stage IIa), immunoreacted with anti vGluT1 (red) and anti TH (green) antibodies. Bar = 10 μm.

## Data Availability

All data generated or analyzed during this study are included in this published article [and its supplementary information files].

## Abbreviations

α-Syn: α-Synuclein (α-Syn)
APP: Amyloid precursor protein
CNS: Central nervous system
DAG: Diacylglycerol
DAT: Dopamine transporter
ICC: Immunocytochemistry
IHC: Immunohistochemistry
NF: Neurofilament
PD: Parkinson’s disease
PI: Phosphatidyl inositol
PI4,5P_2_: Phosphatidylinositol 4,5-bisphosphate
PIP: Phosphoinositides
PIPKI: PIP-5 kinase
PLC: Phospholipase C
PM: Plasma membrane
Sj-1: Synaptojanin-1
SNc: Substantia nigra *pars compacta*
TH: Thyrosine hydroxylase
vGluT1: Vesicular glutamate transporter 1
VTA: Ventral tegmental area
WMT: White matter tract
