# Supplementary material for: A role for α-Synuclein in axon growth and its implications in corticostriatal glutamatergic plasticity in Parkinson’s disease
Source: Mol Neurodegener. 2020 Mar 30;15:24. doi: 10.1186/s13024-020-00370-y (PMC7104492; doi:10.1186/s13024-020-00370-y)
Supplement: Supplementary file 1 — Additional file 1. Table S1. Post mortem human brains. Table S2. list of antibodies. [file 13024_2020_370_MOESM1_ESM.docx]

Supplementary information

Table 1. Post mortem human brains

| source | case ID | gender | age | age at diagnosis | duration | PMl (h) | Braak/ Unified stage |
| --- | --- | --- | --- | --- | --- | --- | --- |
| **PD** | | | | | | | |
| Banner Sun Health Research Institute | 99-09 | F | 77 | 56 | 21 | 2.66 | unified IIa |
|  | 05-17 | F | 84 |  | 22 | 2 | unified IIa |
|  | 04-17 | M | 83 | 75 | 8 | 2 | unified IIa |
|  | 00-09 | M | 64 | 49 | 15 | 4 | unified IIb |
|  | 00-24 | M | 72 | 55 | 17 | 10 | unified IIb |
| UK brain bank | PD084 | M | 86 | 78 | 9 | 3 | Braak 5 |
|  | PD093 | F | 81 | 67 | 14 | 22 | Braak 6 |
|  | PD099 | M | 82 | 72 | 11 | 10 | Braak 6 |
|  | PD104 | M | 75 | 50 | 25 | 15 | Braak 5 |
|  | PD109 | M | 72 | 66 | 6 | 9 | Braak 4 |
|  | PD117 | F | 77 | 46 | 31 | 6 | Braak 5 |
| **Control** | | | | | | | |
| Banner Sun Health Research Institute | 09-57 | M | 80 |  | n.a. | 3.5 |  |
|  | 10-13 | M | 85 |  | n.a. | 3 |  |
|  | 15-60 | M | 82 |  | n.a. | 3.33 |  |
| UK brain bank | PDC094 | F | 80 |  |  | 63 |  |
|  | C022 | F | 69 |  |  | 33 |  |
|  | C032 | M | 88 |  |  | 22 |  |
|  | C045 | M | 77 |  |  | 22 |  |

PMI, post mortem interval in hours (h).

**Table 2. list of antibodies**

| **Antigen** | **Clone and source** | **Test** | **Dilution and blocking** | **Antigen retrieval** |
| --- | --- | --- | --- | --- |
| NF-200 | N4142, PC  Sigma-Aldrich, Rehovot, Israel | IHC | 1:200  o.n 4^0^C, 10% NGS | 95^0^C, 20’ |
|  |  | ICC | 1:200  2h RT,1% BSA in PBS |  |
| Non-P  NF-H | SMI-32, MC  Covance Inc., Princeton, NJ, USA | IHC | 1:1000  2 h RT, 5% NGS | 110^0^C, 15’ |
| P NF-H | SMI-31, MC  Covance Inc., Princeton, NJ, USA | IHC | 1:1000  2h RT, 5% NGS | 110^0^C, 15’ |
| APP | 22C11, MC  Chemicon | IHC | 1:2000  o.n 4^0^C, CAS-block | 110^0^C, 15’ |
| TH | TH-2, MC  Sigma-Aldrich, Rehovot, Israel | IHC | 1:3000  o.n. 4^0^C, CAS-block | 110 ^0^C, 15’ |
|  |  | WB | 1:10,000 |  |
| vGluT1 | AB#5905, PC  Millipore  Rosh-Ha'ayin, Israel | IHC | 1:1000  2 h 37^0^C, 10% NGS, 0.3% Tx-100 | 110 ^0^C, 10’ |
| PI4,5P_2_ | Z-P045, MC  Echelon Biosciences UT, USA | IHC | 1:100  o.n 4 ^0^C CAS-block | 95 ^0^C , 10’ |
|  |  | ICC | 1:200; 2h RT 1% BSA in PBS |  |
|  |  | FACS | 1:200; 1.5 h 4^0^C, 1% BSA in PBS |  |
| α-tubulin | YL1/2 MC,  Serotec | ICC | 1:1000 2h RT, 1% BSA in PBS |  |
| α-Syn | MJFR1, MC  ab138501, abcam | ICC | 1:2000; 2h RT, 1% BSA in PBS |  |
|  |  | FACS | 1:2000 1.5 h 4^0^C 1% BSA in PBS |  |
|  |  | WB | 1:20,000 o.n 4^0^C |  |
| α-Syn | BD Transduction Labs | IHC | 1:1000 |  |
| α-Syn | C20, PC  Santa Cruz. | ICC | 1:500 o.n 4^0^C 1.5%BSA in PBS |  |
| α-Syn | Syn303, PC  Covance Inc., Princeton, NJ, USA | IHC | 1:3000 ; o.n 4^0^C 1.5%BSA in PBS | 95 ^0^C, 10’ |
| Filament α-Syn | MJFR-14-6-4-2  Abcam | IHC | 1:3000; 2.5 hours at RT, CAS-block | 110 ^0^C, 13’ |
| Synaptophysin | Clone Z66, PC  Thermo Sceintific | WB | 1:250 |  |
| DAT | 6-8D6 (sc-32259)  Santa Cruz | WB | 1:300 |  |
| β-actin | ac-15, MC, Sigma-Aldrich | WB | 1:5000 30 min RT |  |
| GAP-43 | AB5220, PC  Millipore  Rosh-Ha'ayin, Israel | ICC | 1:1000 2h RT, 1% BSA in PBS |  |
| Nir2 | AB22823  Millipore  Rosh-Ha'ayin, Israel | WB | 1:5000 90 min RT |  |

*, in 10 mM citrate buffer pH 6.0. PC, polyclonal; NDS, normal donkey serum; MC, monoclonal; NGS, Normal Goat serum, in Tris-Cl pH 7.3, 0.3% TX-100; CAS-block (Thermo Fisher) 10 min at RT; NDS, normal donkey serum (Jackson ImmunoResearch, ME, USA).
