# Supplementary material for: A role for α-Synuclein in axon growth and its implications in corticostriatal glutamatergic plasticity in Parkinson’s disease
Source: Mol Neurodegener. 2020 Mar 30;15:24. doi: 10.1186/s13024-020-00370-y (PMC7104492; doi:10.1186/s13024-020-00370-y)

Supplementary Fig. 1

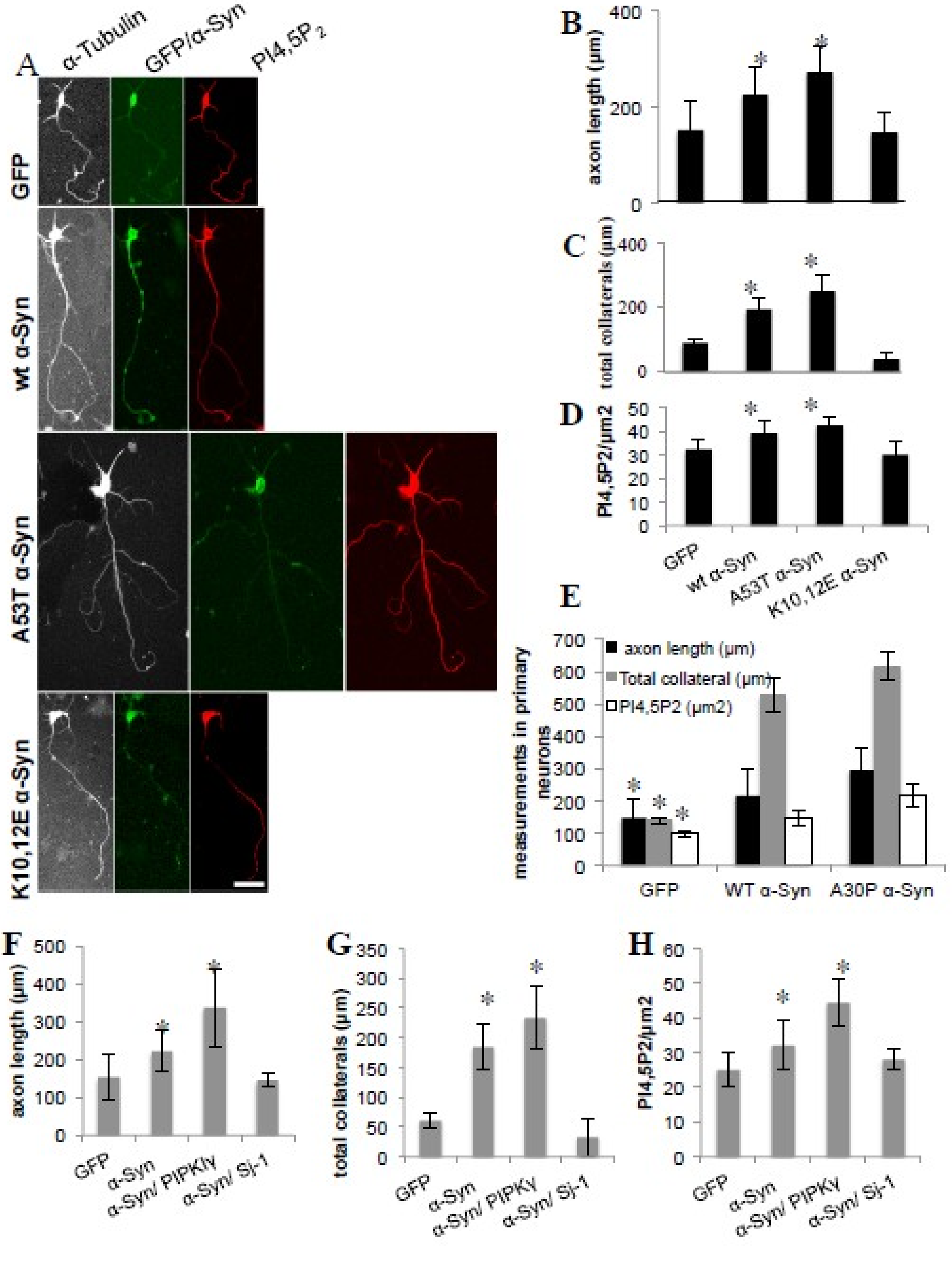

Supplementary Fig. 2

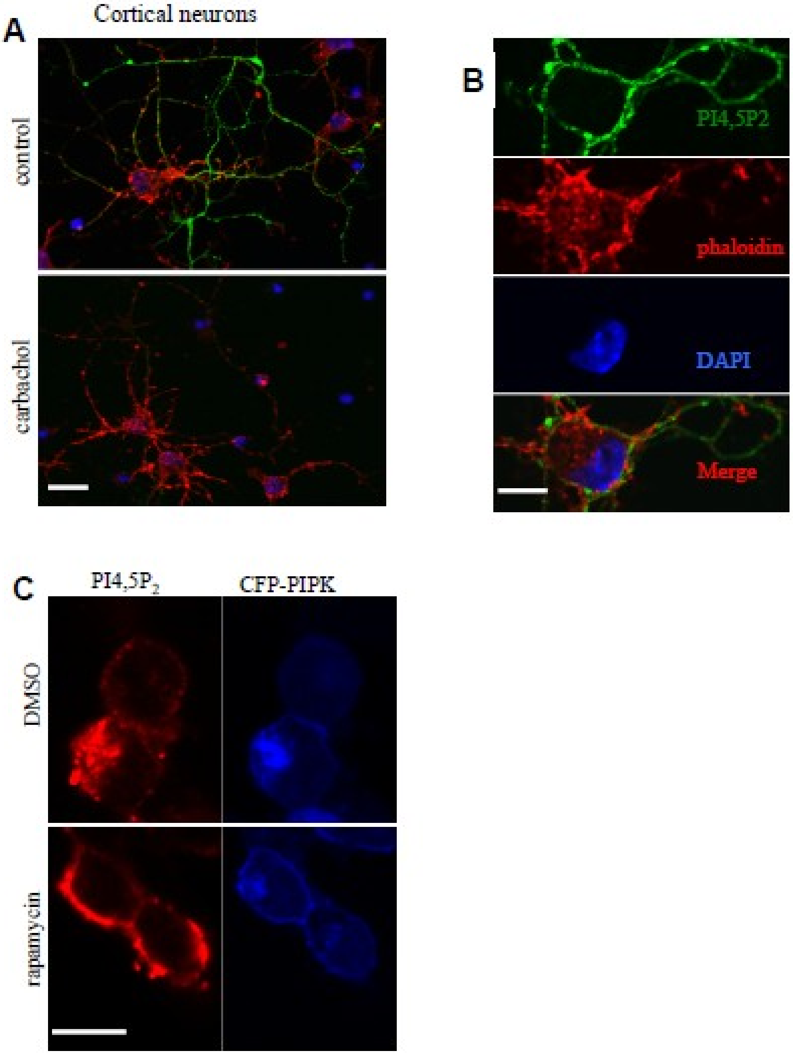

Supplementary Fig. 3

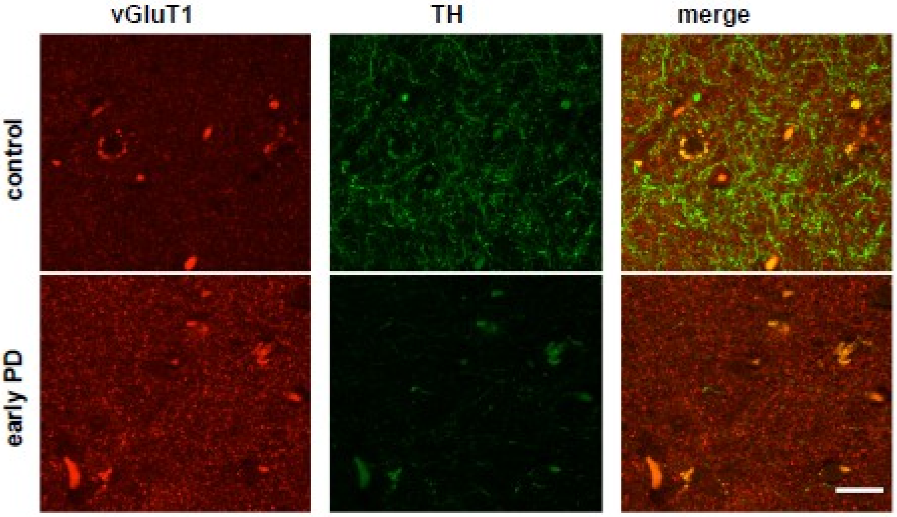

Supplement: Supplementary file 2 [file 13024_2020_370_MOESM2_ESM.docx]
